# Large genomic, functional, and phenotypical diversity of *Janthinobacterium* associated with Atlantic salmon fry

**DOI:** 10.1093/femsmc/xtaf015

**Published:** 2025-10-29

**Authors:** Eirik Degré Lorentsen, Eva C Sonnenschein, Alexander W Fiedler, Ingrid Bakke

**Affiliations:** Norwegian University of Science and Technology, 7491 Trondheim, Norway; Swansea University, Swansea, SA2 8PP Wales, United Kingdom; Norwegian University of Science and Technology, 7491 Trondheim, Norway; Enova, 7010 Trondheim, Norway; Norwegian University of Science and Technology, 7491 Trondheim, Norway

**Keywords:** *Janthinobacterium*, *Salmo salar*, yolk sac fry, comparative genetics, phylogeny, violacein

## Abstract

Members of the genus *Janthinobacterium* are widespread and found in soil and freshwater ecosystems, but also in the skin of humans, fish, and amphibians. They are known for producing violacein, and they typically have antifungal properties. In amphibians, *Janthinobacterium* spp. protect their hosts against fungal infections. We examined the diversity of five *Janthinobacterium* strains isolated from the skin and rearing water of Atlantic salmon fry by phenotypic characterization and comparative genomics. Although their 16S rRNA gene sequences were almost identical, their phenotypes were highly dissimilar, and only two of the species consistently produced violacein. Genomic analyses revealed that they represented five species, and phylogenetic analysis suggested that only one was closely related to a previously described species (*Janthinobacterium tructae*^T^). All strains possessed the *Janthinobacterium* quorum sensing system, while three harbored genes of the AHL QS system. They had great potential for producing secondary metabolites, and one carried putative genes of the antibiotic tropodithietic acid, previously described in the marine *Phaeobacter*. Interestingly, they all carried putative genes for heterotrophic carbon fixation. Furthermore, they had the genetic potential for chemotaxis and motility; compatible with a host-associated lifestyle. Gnotobiotic experiments confirmed that they were able to colonize yolk sac fry of Atlantic salmon.

## Teaser

Five strains of *Janthinobacterium* with highly similar 16S rRNA sequences, but diverse genome sequences and phenotypes were isolated from fry and rearing water of Atlantic salmon.

## Introduction

*Janthinobacterium* is a genus of Gram-negative (Osullivan et al. 1990) and aerobic (Deley et al. 1978) betaproteobacteria commonly isolated from soil and freshwater (Haack et al. 2016), including extreme environments, such as Antarctica (Shivaji et al. 1991), Arctic seawater (Alonso-Saez et al. 2014), and the Himalayas (Kumar et al. 2018). *Janthinobacterium* has also been found to be associated with a diverse range of hosts, such as different fish species (Wang et al. 2017, Oh et al. 2019, Emam et al. 2020), including Atlantic salmon (Gajardo et al. 2016, Wang et al. 2018, Uren Webster et al. 2020, Fiedler et al. 2023), the fish parasite *Pseudoterranova decipiens* (Saelens and Houf 2022), sponges (Belikov et al. 2021), ticks (Galaviz-Silva et al. 2022), plants (Xia et al. 2021, Yin et al. 2021), amphibians (Brucker et al. 2008, Becker et al. 2009, Bresciano et al. 2015), giant pandas (Yang et al. 2017), and humans (Grice et al. 2008, Gonzalez et al. 2021, Xu et al. 2021). Interestingly, *Janthinobacterium* appears to be an important taxon in the microbiomes of highly diverse skin tissues, such as human (Grice et al. 2008), salmon (Wang et al. 2018, Fiedler et al. 2023), and amphibian (Brucker et al. 2008, Becker et al. 2009, Harris et al. 2009, Bresciano et al. 2015) skin. It has previously been shown that amphibians carrying *Janthinobacterium lividum* on their skin have increased survival during fungal infection (Brucker et al. 2008, Becker et al. 2009, Harris et al. 2009). Antagonism against fungi appears to be a typical characteristic of *Janthinobacterium* (Brucker et al. 2008, Harris et al. 2009, Wang et al. 2012, Haack et al. 2016, Yin et al. 2021). Experiments using *J. lividum* strains isolated from the skin of the salamander *Hemidactylium scutum* as a probiotic treatment against the fungal infection *Batrachochytrium dendrobatidis* on frogs (Harris et al. 2009) and salamanders (Becker et al. 2009) showed promising results. Moreover, *Janthinobacterium* strains associated with salamander skin produced metabolites in sufficient concentrations to hinder growth of the fungus *B. dendrobatidis* (Brucker et al. 2008). Also, *J. lividum* has been shown to ameliorate the effects of fungal *Trichophyton rubrum* infections on human skin (Ramsey et al. 2015).

Genomic studies of *Janthinobacterium* have demonstrated remarkable adaptations to their environment. For example, they exhibit a high potential for producing a diversity of secondary metabolites (Haack et al. 2016, Friedrich et al. 2020, Belikov et al. 2021, Wu et al. 2021). Many *Janthinobacterium* strains are known to produce the purple pigment violacein (Kimmel and Maier 1969, Deley et al. 1978, Pantanella et al. 2007, Wu et al. 2021). This compound has been found to possess antibacterial (Lichstein and Vandesand 1945, Pantanella et al. 2007, Wang et al. 2012, Asencio et al. 2014), antioomycete (Wang et al. 2012, Yin et al. 2021), and antitumoral activities (De Azevedo et al. 2000), and has often been assumed to explain the antifungal properties of *Janthinobacterium* strains (Brucker et al. 2008, Becker et al. 2009, Wang et al. 2012). However, Haack et al. (2016) found that the antifungal properties of *Janthinobacterium* were reduced by knocking out the *Janthinobacterium* quorum sensing (JQS) system, and that the antifungal properties were not primarily linked to violacein. Furthermore, they found that the fungal inhibition was dependent on the chitin degradation product *N*-acetyl-d-glucosamine (NAG) (Haack et al. 2016). Genome sequencing has indicated that most members of the *Janthinobacterium* genus possess a quorum sensing (QS) system called JQS system, which regulates the expression of the violacein operon (Hornung et al. 2013, Haack et al. 2016, Wu et al. 2021). The JQS system is also involved in the regulation of genes encoding secondary metabolites, Type VI secretion systems, Flp type pilus, including genes involved in the inhibition of the fungal plant pathogen *Fusarium graminearum* (Haack et al. 2016). JQS includes three genes: the autoinducer synthase *jqsA*, the sensor kinase *jqsS*, and the regulator protein *jqsR* (Haack et al. 2016). It is similar to the QS systems found in *Vibrio cholerae* and *Legionella pneumophila*, which are involved in regulating host–pathogen interactions (Tiaden and Hilbi 2012, Simon et al. 2015). Furthermore, *Janthinobacterium* strains have been shown to degrade chitin by secretion of chitinases (Haack et al. 2016). The chitin degradation product NAG seems to have a role in the JQS regulatory circuit, which in return may be involved in antifungal activity (Haack et al. 2016). Valdes et al. (2015) found that the strain *J. lividum* MTR possessed capnophilic properties, i.e. its growth was promoted by heightened CO_2_ concentrations. They suggested that this property was linked to the functional potential for heterotrophic carbon fixation through the glyoxylate cycle (Valdes et al. 2015).

In a collection of bacterial strains, previously isolated from salmon fry in our research group, we identified five strains that were taxonomically assigned to the genus *Janthinobacterium*. Of these, three were isolated from skin of fry in a commercial recirculating aquaculture facility, whereas two were isolated from the rearing water of small-scale flasks with yolk sac fry. In this study, we aim to assess the diversity of these *Janthinobacterium* strains by genotypical and phenotypical characterization, including genomic similarity, genomic potential for secondary metabolite production, and potential for violacein production. Furthermore, we examine their ability to colonize Atlantic salmon yolk sac fry.

## Material and methods

### Isolation of bacterial strains

The three *Janthinobacterium* strains 3.108, 3.109, and 3.116 were isolated from the skin of Atlantic salmon sampled from one rearing tank with fry of an average weight of 21.8 g in a commercial flow-through system. The isolation of 3.108 has been reported previously (Fiedler et al. 2023), and 3.109 and 3.116 were isolated as described therein. These isolates were the only ones identified as *Janthinobacterium* by 16S rRNA sequencing (see below) among a total of around 75 isolates collected from the gut and skin of salmon fry. The two *Janthinobacterium* strains, pbA and pbB, were isolated from the rearing water of yolk sac fry of an aquaculture strain of Atlantic salmon (supplied by AquaGen AS) reared in tissue culture flasks as follows: 100 µl of fish rearing medium (SGM) were plated out on sterile tryptic soy agar (TSA, Millipore®) plates using glass beads. The agar plates were incubated at room temperature for several days. Dark purple single colonies were transferred to new TSA plates to isolate pure strains. All the isolates were identified as *Janthinobacterium* by 16S rRNA sequencing (see below).

### DNA extraction

To extract genomic DNA, the five strains were cultivated overnight in 250 ml baffled Erlenmeyer flasks with 25 ml liquid tryptic soy broth (TSB, Millipore®) at 300 rpm and 22°C. For each strain, 3 ml of bacterial culture was centrifuged (13 000 × *g*, 1 min). The supernatant was removed, and the pellet was used for DNA isolation using the DNeasy® Powerlyzer PowerSoil® kit (Qiagen), following the manufacturer’s protocol. The concentration and quality of the extracted genomic DNA was measured using a NanoDrop™ One (Thermo Scientifc™). The concentration of the extracted DNA was between 32 and 59.5 ng/μl, and the A260/A280 ratios were in the range 1.89–1.90, and the A260/A230 ratios were in the range 1.95–2.03. Thus, the DNA quality was sufficient for genome sequencing.

### Amplification, sequencing, and classification of the 16S rRNA gene

To amplify the 16S rRNA gene of the strains, Polymerase Chain Reaction (PCR) was run for 37 cycles (98°C 15 s, 55°C 20 s, and 72°C 20 s) in a T100™ Thermal Cycler (Biorad) using EUB8.F (5′-AGA GTT TGA TCM TGG CTC AG-3′) (Weisburg et al. 1991) and 1492.R (5′-TAC GGY TAC CTT GTT ACG ACT T-3′) (Weisburg et al. 1991) as primers at a final concentration of 0.3 µM per primer with 0.2 mM of each dNTP (VWR), 1.5 mM MgCl_2_, 0.012 units/µl of Phusion Hot Start DNA polymerase and HF reaction buffer from Thermo Scientific™. Genomic DNA was used as a template (1 µl of genomic DNA to a reaction mix of a total of 25 µl). After assessing the quality of the PCR products with agarose gel electrophoresis, the PCR products were purified using the QIAquick® PCR Purification Kit (Qiagen) following the producer’s protocol. The purified PCR products were sequenced using both PCR primers as sequencing primers at Eurofins Genomics with Sanger sequencing. The 16S rRNA gene sequences were assembled using the Clone Manager software (version 9.51). The RDP Classifier tool (Wang et al. 2007) was used to assign taxonomy to the sequences.

### Growth conditions of bacteria and colony morphology

The *Janthinobacterium* strains were generally cultivated in TSB or on TSA at RT (21°C–22°C). To prevent the formation of bacterial aggregates in liquid media, 0.1% of Tween-80 (Sigma-Aldrich) was added to TSB, baffled Erlenmeyer flasks were used, and the shaking speed was set to 300 rpm. To investigate if the *Janthinobacterium* strains could utilize chitin or mucin as the sole carbon source, M9 medium (10.5 g/l, Sigma-Aldrich) with 2 mM MgSO_4_ and 1.5% agar was prepared with either mucin from porcine stomach (4 g/l, Sigma-Aldrich) or colloidal chitin (0.4%). Plates without other carbon sources than agar were used as controls. Colloidal chitin was prepared as described previously (Mølmen 2021). In brief, 3 g of chitosan (Chitinor) was dissolved to 1% (w/v) in acetic acid (0.33 M) and transferred to a dialysis bag (12–14 kDa MWCO, Spectrapor®). The dialysis was conducted in 7 l of water with 50 mM NaCl, replacing the water after >4 h five times before the final dialysis against ion-free water. This water was changed five times, making the total number of water changes 10, before the content of the dialysis bag was freeze-dried in liquid N_2_ using a rotavapor (Christ Alpha 1–4 LO). The colloidal chitin was dissolved in MilliQ water before media preparation. The colony morphology of the five strains when grown on TSA plates were assessed by visual inspection.

### Assessing the potential for violacein production

The experiments for assessing the potential violacein production of the *Janthinobacterium* strains were a part of a master’s project, and were performed only for the strains 3.108 and 3.116 (Lorentsen 2020). The experiments were performed by cultivating strain 3.108 and 3.116 on various growth media at different temperatures (22°C–28°C). The growth media applied were TSA, Luria–Bertani agar (LA), LA with 2% glycerol, extracellular polymeric substance medium [EPS medium (Raza et al. 2011, Rütering et al. 2017)], and EPS + sucrose (EPS medium with 3% sucrose instead of glucose, Table S1), and all media were made with 1% or 1.5% agar. Most of the bacterial cultivations were performed at room temperature and on solid media, unless stated otherwise. The plates were incubated for 3–7 days, and regularly inspected for the growth of purple colonies (Lorentsen 2020). Strains forming purple colonies were then grown in liquid medium, and the potential violacein production was observed as a purple ring occurring on the interface between air and liquid medium. After cultivation for 1 week, the purple interface was carefully collected and dissolved in 3 ml ethanol (96%), and centrifuged (18 000 × *g*, 1 min). To verify that violacein was responsible for the purple color, the maximum absorbance was measured using a V-1200 spectrophotometer (VWR) on wavelengths between 300 and 800 nm, as described by Mølmen (2021). Violacein is known to have maximum absorbance at ~570 nm (Rettori and Durán 1998).

### Test of antibiotic susceptibility

To test the antibiotic susceptibility of the *Janthinobacterium* strains, LA-plates containing antibiotics were prepared. The susceptibility was tested for ampicillin (100 µg/ml), erythromycin (50 µg/ml), kanamycin (50 µg/ml), streptomycin (25 µg/ml), spectinomycin (100 µg/ml), chloramphenicol (50 µg/ml), and tetracycline (10 µg/ml). The *Janthinobacterium* strains were plated out on LA plates containing antibiotics and incubated for ~5 days before the growth was visually inspected. *Escherichia coli* K12 MG1655 was included as a control for antibiotic susceptibility.

### Genome sequencing of the five *Janthinobacterium* strains

The previously extracted genomic DNA (30 μl) of the five *Janthinobacterium* strains, 3.108 (32.0 ng/µl), 3.109 (56.8 ng/µl), 3.116 (46.4 ng/µl), pbA (59.4 ng/µl), and pbB (48.3 ng/µl) was sent to Novogene for whole genome sequencing using an Illumina Novaseq 6000 instrument. Genomic analysis, assembly, and annotation was mainly performed through the kbase platform using the default parameters (Arkin et al. 2018). The sequencing files were quality assessed using FastQC v0.11.9 (https://www.bioinformatics.babraham.ac.uk/projects/fastqc/) and trimming was performed with Trimmomatic v0.36 (Bolger et al. 2014). Genome assembly was performed using SPAdes v3.15.3 (Prjibelski et al. 2020). CheckM v1.0.18 (Parks et al. 2015) was used to ensure the quality of the assembly. The annotation was performed using Prokka v1.14.5 (Seemann 2014). The sequence similarity of the *Janthinobacterium* strains was determined with FastANI (Jain et al. 2018) to calculate the average nucleotide identity (ANI). Additionally, *in silico* DNA–DNA hybridization (isDDH) was done by using the Type (strain) Genome Server (TYGS) (Meier-Kolthoff and Göker 2019). Furthermore, species identification through pubMLST was used to further verify the species demarcation of the strains (Jolley et al. 2018). A pangenome of the five newly sequenced *Janthinobacterium* strains and the seven genomes of *Janthinobacterium* type strains (based on TYGS), namely *Janthinobacterium agaricidamnosum* DSM 9628^T^ (accession number: GCA_000723165.1), *Janthinobacterium aquaticum* FT58W^T^ (accession number: GCA_009208565.1), *J. lividum* EIF1^T^ (accession number: GCA_013372045.1), *Janthinobacterium psychrotolerans* S3-2^T^ (accession number: GCA_001677885.1), *Janthinobacterium rivuli* FT68W^T^ (accession number: GCA_009208735.1), *Janthinobacterium tructae* SNU WT3^T^ (accession number: GCA_006517255.1), and *Janthinobacterium violaceinigrum* FT13W^T^ (accession number: GCA_009208555.1) were constructed using anvi’o (version 7.1; Eren et al. 2021), following the anvi’o workflow for pangenomic analysis (https://merenlab.org/2016/11/08/pangenomics-v2/). The pangenome was separated into the core genome, including genes represented in all strains, singletons, including genes only found in one of the strains, and the shell genes, which are the remaining genes. Scripts used for constructing the pangenome are publicly available through GitHub (https://github.com/eidelo/Genomic-diversity-Janthinobacterium). Additionally, a phylogenetic tree including all currently available refseq strains of *Janthinobacterium* in the NCBI database (Table S2) was constructed using the pyPGCF software (Nikolaidis et al. 2024). First, the species demarcation module used FastANI (Jain et al. 2018) to calculate pairwise ANI between the genomes and Markov Clustering (Van Dongen 2008) to cluster them into species (Nikolaidis et al. 2024). The orthologues module uses reciprocal BLAST/DIAMOND to identify orthologue genes (Nikolaidis et al. 2024). These orthologues were used to find the core genome using the core module. The core genes were used as input in the phylogenetic module, which does an alignment of the orthologous groups with MUSCLE (Edgar 2004) and filtering with Gblocks (Castresana 2000, Talavera and Castresana 2007). The phylogenetic module utilizes these as input when constructing a maximum-likelihood phylogenetic tree with IQTree2 (Minh et al. 2020) using 1000 bootstraps with SH-like aLRT (Guindon et al. 2010) and ModelFinder (Kalyaanamoorthy et al. 2017) to find the best model for the tree construction, which was found to be Q.plant+F+I+G4. Scripts used for constructing the phylogenetic tree are publicly available through GitHub (https://github.com/eidelo/Genomic-diversity-Janthinobacterium). The tree was visualized using the interactive tree of life (Letunic and Bork 2021). A tree containing *Oxalobacter formigenes* OxB (GCA_027158485.1) as an outgroup was first constructed. This tree was used to find the most distantly related *Janthinobacterium* strain to select as an outgroup in the final tree. To compare the evolution of the 16S rRNA gene and a set of genomic core genes, we constructed phylogenetic trees for the seven *Janthinobacterium* type-strains and the strains isolated in this study with *O. formigenes* OxB as outgroup. The phylogenetic tree for the genomic core genes was constructed as described above. The phylogenetic tree based on the 16S rRNA gene sequences was constructed using Molecular Evolutionary Genetics Analysis Version 12 for adaptive and green computing (MEGA12) (Kumar et al. 2024). First, the 16S rRNA gene sequences were aligned using MUSCLE (Edgar 2004), before trimming off sequences in the 3′ and 5′ prime ends that were not present for all the strains. A maximum-likelihood tree was constructed using the Tamura-Nei model and 1000 bootstraps (Tamura and Nei 1993). The phylogenetic trees were visualized using the interactive tree of life platform (Letunic and Bork 2021). Furthermore, VSEARCH was used to make an alignment of the 16S rRNA sequences, and identify sequence similarities (Rognes et al. 2016). Screening for secondary metabolites was performed with antiSMASH (v7.1.0; Blin et al. 2023) with default parameters through their web-platform. As the genomes were sequenced to contig level, there is a chance that some gene clusters encoding secondary metabolites could be fragmented on several contigs if present at the end of a contig; thus, the placement of duplicate genes on the contigs was therefore checked manually. The fraction of the genomes designated to biosynthetic gene clusters (BCGs) was calculated according to Paulsen et al. (2019). Geneious Prime 2023.0.1 (https://www.geneious.com) was used to map genomes to reference sequences and to identify specific genes in the genomes. Protein–Protein BLASTp (v2.12.0+; Altschul et al. 1997, Camacho et al. 2009) was performed for AHL-related genes (LuxI; accession number: OEZ51813.1), LuxR (accession number, NCBI protein database: OEZ51814.1), and LuxI (accession number, NCBI protein database: OEZ48213), TDA genes (accession numbers, NCBI protein database: AUQ60543.1, AUQ60542.1, AUQ60541.1, AUQ60540.1, AUQ60539.1, and AUQ60526.1). To annotate carbohydrate-active enzymes, the dbCAN3 server was used (Zheng et al. 2023). To screen for phage sequences in the bacterial genomes, PHASTER was used through their web-platform with default parameters (Zhou et al. 2011, Arndt et al. 2016). ARTS: Antibiotic Resistant Target Seeker Version 2 (Alanjary et al. 2017, Mungan et al. 2020), the Center for Genomic Epidemiology’s ResFinder (4.6) tool (Camacho et al. 2009, Clausen et al. 2018, Bortolaia et al. 2020), and the Comprehensive Antibiotic Resistance Database (CARD) (Alcock et al. 2023) were used to screen the genomes for antibiotic resistance genes using default parameters through their web-platform. The Microscope platform was used to compare the virulence of the *Janthinobacterium* strains using the Virulence factor database (Liu et al. 2022). The *Janthinobacteria* genomes are available at NCBI under the BioProject: PRJNA962300, with the following accession numbers: strain 3.116 (JASAUM000000000), strain 3.109 (JASAUN000000000), strain 3.108 (JASAUO000000000), strain pbB (JASAUP000000000), and strain pbA (JASAUQ000000000).

### Colonization of Atlantic salmon yolk sac fry by the *Janthinobacterium* strains

To investigate the ability of the five *Janthinobacterium* strains to colonize Atlantic salmon fry, we performed two independent experiments (Exp.1 and Exp.2), where we exposed germ-free Atlantic salmon eggs to the *Janthinobacterium* strains in small-scale rearing flasks. Around 1 week before hatching, we added the strains separately to the fish flasks (i.e. mono-associations), aiming at a final concentration of 10^5^ cells per ml. For Exp.1, one flask was used per strain, while for Exp.2, four replicate flasks were used for each strain. The experiments were maintained until 14 and 19 days after hatching for Exp. 1 and Exp.2, respectively, at which the colonization success for the strains was evaluated by colony-forming unit (CFU) analysis. Exp. 1 was conducted as part of a master thesis, and the experiment and preliminary results have been described in the thesis (Hailu 2023).

For Atlantic salmon yolk sac husbandry, the procedure described by Gómez de la Torre Canny et al. (2023) was followed. The eggs were provided from AquaGen AS’s facility in Hemne or Steigen (Norway) at ~80% developmental status. They were immediately placed in a dark room at 6°C in Petri dishes (13.5 cm Ø) and covered with Salmon Gnotobiotic Medium (SGM) (0.5 mM MgSO_4_, 0.054 mM KCl, 0.349 mM CaSO_4_, and 1.143 mM NaHCO_3_) (Gómez de la Torre Canny et al. 2023). The eggs were sterilized 24 h after arrival at our laboratory following the procedure described by Gómez de la Torre Canny et al. (2023), except for a change in the oxolinic acid concentration, which was reduced from 75 to 37.5 mg/l. After treatment with an antibiotics cocktail and disinfection with buffodine (FishTech AS), the eggs were transferred to 250 ml tissue cell-culture flasks containing 100 ml SGM, at a density of 11–14 eggs per flask. Three days after the disinfection, a sterility test of the rearing water was performed as described by Fiedler et al. (2023). Three times a week throughout the experimental period, 60% of the SGM was exchanged with new SGM to maintain good water quality. When observed, dead fish were removed. The fish flasks were kept at a water temperature of 6.6 ± 0.4°C. On day 4 or 5 after disinfection, the germ-free eggs were exposed to the *Janthinobacterium* strains through the rearing water. Overnight cultures of *Janthinobacterium* strains 3.108, 3.109, 3.116, pbA, and pbB were made by inoculating baffled Erlenmeyer flasks containing TSB (25 ml, with 0.1% Tween-80) with 3% growing *Janthinobacterium* culture. The cultures were incubated overnight at 22°C with shaking at 300 rpm. The *Janthinobacterium* cultures were added separately to the flasks with germ-free eggs 4 or 5 days after disinfection (for Exp. 1 and Exp. 2, respectively) and 100 ml of SGM. We aimed at a final concentration of about 1 × 10^5^ CFU/ml. The bacterial concentration added to the fish flasks was estimated based on OD_600_ measurements. In addition, we determined the initial cell density by flow cytometry, as described below. For Exp.1, this was done for samples of the bacterial cultures prior to the addition to fish flasks, while for Exp.2, it was performed for samples of the rearing water directly after the addition of the bacteria. For Exp.1 and Exp.2, the eggs hatched 8 and 7 days post exposure to bacteria, respectively, where the day of hatching was defined as the day at which more than 60% of the eggs had hatched.

The concentration of bacterial cells in the rearing water of each flask at the end of the experiments was determined using flow cytometry, as described below. In addition, the bacterial concentrations in the rearing water and the bacterial loads associated with the Atlantic salmon yolk sac fry at the end of the experiments were assessed using CFU analysis. From each rearing flask, 3 or 1 fish were sampled (Exp.1 and Exp. 2, respectively). For a total of 15 or 20 fish for Exp. 1 and Exp. 2, respectively. Individual fish were first euthanized by a tricaine overdose [5.2 g tricaine in 27.3 ml 1 M Tris buffer (pH 9) and up to 1 l of milliQ water, sterile filtrated], rinsed in sterile SGM, and added to tubes with zirconium beads (1.4 mm, Bertin Technologies) and 400 µl sterile SGM. The fish were then homogenized using a Precellys 24 (Bertin Instruments, 2500rpmX30 secondsX2). For the rearing water, two or one sample of 400 µl was collected from each rearing flask in tubes with zirconium beads (1.4 mm, Bertin Technologies) for Exp.1 and Exp.2, respectively. Thus, giving a total of 10 water samples for Exp.1 and 20 water samples for Exp.2. The water samples were homogenized using a Precellys 24 (Bertin Instruments, 2500rpmX30 secondsX2). For the fish samples of Exp.1, 50 µl of undiluted, 1:10, and 1:100 dilutions of the fish homogenates were plated on TSA plates in triplicate. For the water samples from Exp.1 and both fish and water samples from Exp.2, 50 µl of 1:10, 1:100, and 1:1000 dilutions of the samples were plated on TSA plates in triplicates. The plates were incubated at room temperature for 4 days before counting the number of CFUs. For four of the fish samples, CFU counts for one of the replicate flasks were discarded as the CFU counts were much higher than expected. This was also the case for one of the plate replicates for water samples from Exp.1 for strain 3.108. This was assumed to be due to a mistake during the preparation of the dilution series, and the plates were discarded from the analysis. The data were analysed using R (4.2.2; Team 2014) and the ggplot2 (3.4.2) package. To test for differences in the ability of the strains to colonize yolk sac fry and establish in the rearing water an analysis of variance test (ANOVA) was used (Heiberger 1992 et al. 1992) with a Tukey HSD *post hoc*.

### Flow cytometry

To determine bacterial density in the bacterial cultures used in Exp.1 by flow cytometry, 490 µl from each of the overnight cultures was fixated with glutaraldehyde to a final concentration of 1%. For Exp.2, the bacterial density in the fish-rearing water was determined using flow cytometry at the start of the experiment. For each fish flask, 490 µl water was sampled and fixated using glutaraldehyde to a final concentration of 1%. SYBR green I (10 000x) was diluted using sterile 0.2 µm filtrated phosphate-buffered saline (PBS) (1x) to make a 200x stock. The samples were diluted 1:5 using 0.2 µm filtrated PBS (1x) and stained using the SYBR green I 200x stock to a final concentration of 2x SYBR green I stain. The stained samples were incubated for 15 min at 37°C and vortexed before being analysed using an Attune NxT (ThermoFisher) flow cytometer with a flow rate of 100 µl/min for 1.5 min. The data were collected using the blue laser (488 nm, 50 mW) with detection in BL1 (530/30 nm) and BL3(695/40). The following voltage settings were used; FSC 370 V, SSC 440 V, BL1 290 V, and BL3 440 V. 0.2 µm filtered PBS and SGM were used as negative controls to identify bacterial populations. The flow cytometry data (.fcs files) are available at https://doi.org/10.6084/m9.figshare.28211000.v1

## Results

### rRNA gene-based classification and phenotypic characteristics of the *Janthinobacterium* strains

To classify the five *Janthinobacterium* strains 16S rRNA gene sequencing was conducted. Sequence analysis of the full 16S rRNA gene assigned three of the strains from a collection of bacterial strains originating from the skin and gut of Atlantic salmon to the genus *Janthinobacterium*: 3.108, 3.109, and 3.116. Additionally, we collected two bacterial strains from the rearing water of yolk sac fry of Atlantic salmon, that were also classified to this genus (pbA and pbB). The 16S rRNA gene sequences were highly similar (>99%) between the five strains and to that of the *J. lividum* type strain DSM1522^T^ (Fig. S1). The 16S rRNA gene sequences of pbA and 3.108 were found to be identical.

**Figure 1.**
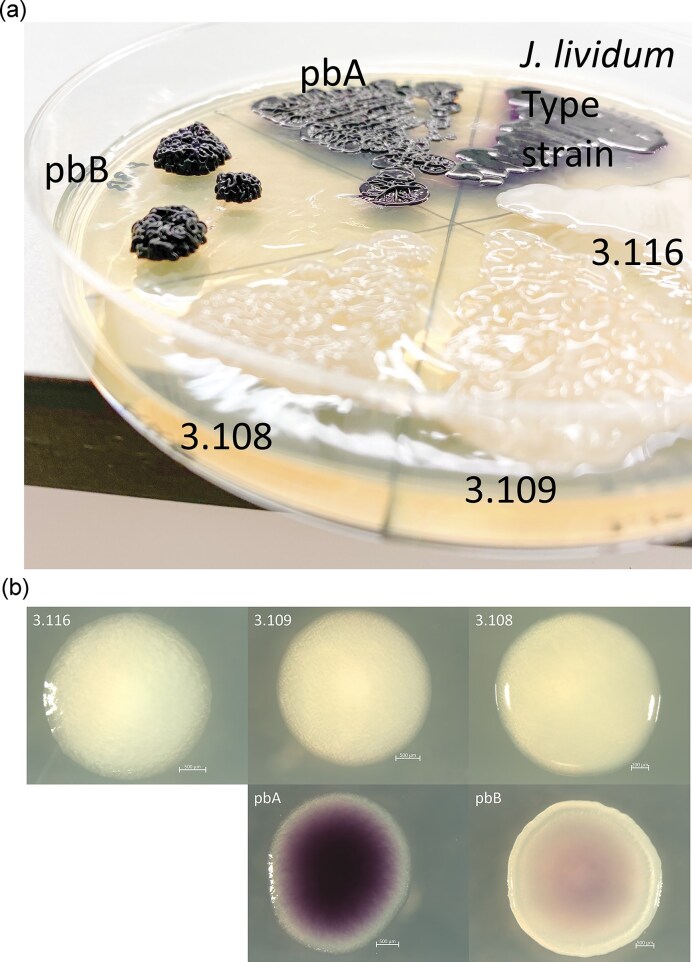
Examples of different colony morphologies of the five *Janthinobacterium* strains. (A) Colonies of the five strains and the *J. lividum* type strain after growing on LA plates (1.5% agar, 2% glycerol) at 18°C for ~2 weeks (Mølmen 2021). (B) The *Janthinobacterium* strains after ~1 week of incubation at room temperature on TSA plates (1.5% agar). All scale bars represent 500 µm, except for the picture of strain 3.108, where the scale bar represents 200 µm.

**Figure 2.**
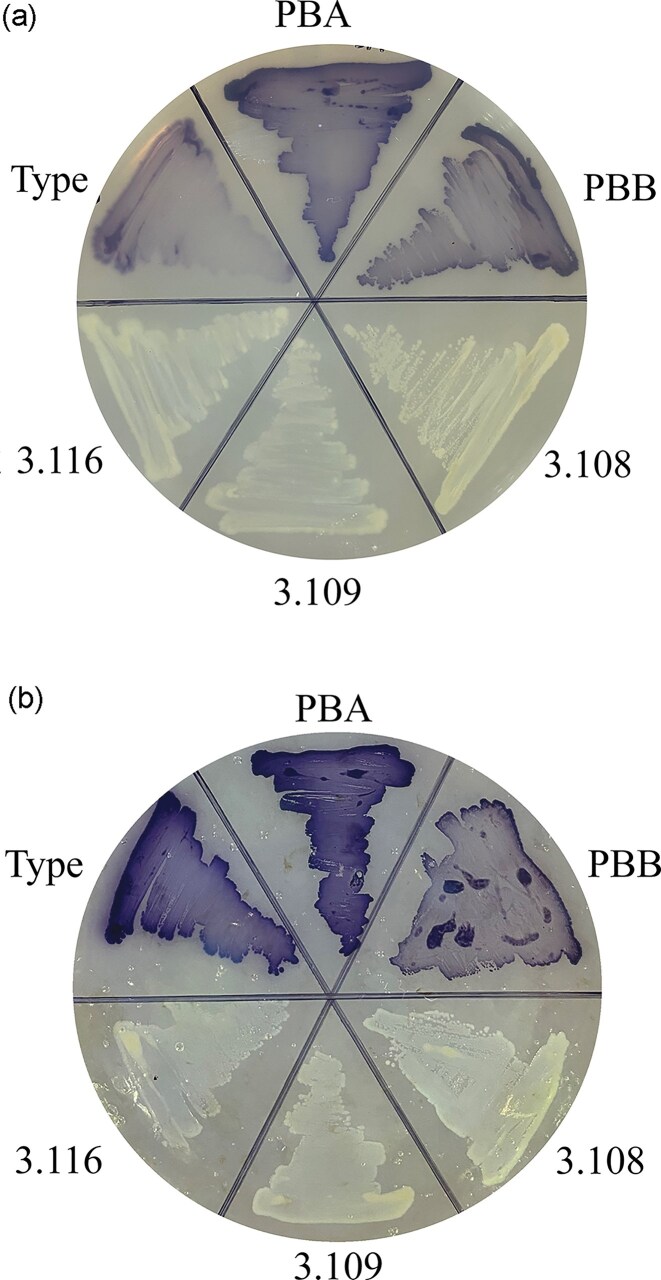
Cultivation of the *Janthinobacterium* strains, pbA, pbB, 3.108, 3.109, and 3.116 and *J. lividum* type strain (DSM1522) on agar media with (A) mucin and (B) chitin as the sole carbon source (Mølmen 2021). No growth was observed for any of the strains when cultivated on the corresponding medium with agar as the only carbon source.

When cultivated on solid media, the five *Janthinobacterium* strains had distinctly different phenotypes (Fig. 1, Table 1). A striking difference was the color: colonies of pbA were always dark purple when grown on agar media (TSA, LA, Glucose yeast agar (GYA), mucin agar, and chitin agar; Figs 1 and 2, Table 1, Table S3), indicating violacein production. Strain pbB was usually observed to have purple colonies (TSA, LA, GYA, mucin agar, and chitin agar; Figs 1 and 2, Table 1, Table S3), but occasionally it was observed to have both white and purple colonies on the same plate (data not shown) and was observed to have a weaker purple colour than pbA (Fig. 1b, Table S3). For strains 3.109 and 3.116, purple colonies were never observed on any agar media, and colonies generally appeared as cream-colored (Fig. 1b, Table 1, Table S3). For strain 3.108, light purple colonies were only observed when grown on LA agar medium with reduced agar concentration (1%) and for growth at 28°C on agar media with glycerol (Fig. S2, Table S3). Also, growth on EPS–sucrose agar media at 28°C occasionally promoted purple pigment formation for this strain (Fig. S2, Table S3). When cultivating pbA and pbB in liquid LB media, a purple ring was formed on the tube wall in the interface between the liquid and air. This was not observed for the other strains. Extraction and spectrophotometric analysis of the purple interface from these liquid cultures resulted in absorption maxima of 576 nm and 574 nm for pbA and pbB, respectively (Fig. S3), which is in accordance with the expected maximum for violacein (Rettori and Durán 1998). This supports the assumption that these two strains produce this compound.

**Table 1.** Characteristics of colonies of the five *Janthinobacterium* strains pbA, pbB, 3.108, 3.109, and 3.116, based on visual inspection after growing on TSA plates at room temperature for 5 days.

Strain	Form/shape	Elevation	Margin	Appearance	Consistency/texture	Colour	Opacity
pbA	Circular	Raised	Entire	Shiny	Creamy/moist	Purple	Opaque
pbB	Circular	Raised	Entire	Shiny	Creamy/moist	Purple/cream white	Opaque
3.108	Circular	Raised	Entire	Shiny	Viscid/sticky	Cream white	Opaque
3.109	Circular	Raised	Entire	Shiny	Creamy but a bit sticky	Cream white	Opaque
3.116	Circular	Raised	Entire	Shiny	Creamy/moist	Cream white	Opaque

In addition to the obvious differences in color, colony morphology and consistency varied between the strains when grown on LA or TSA agar plates (Fig. 1a and b, Fig. S2, Table 1, Table S3). The colonies of 3.108 were stickier than the remaining strains (Table 1). Furthermore, colonies of pbB usually had an undulate margin, while the margin of the other strains was more even (Fig. 1b, Table 1). Colonies of pbB showed a tendency to grow into more raised colonies (Fig. 1a).

To test the chitinoclastic activity and the ability to utilize mucin as a carbon source, we cultivated the strains on agar media with either chitin or mucin as the only carbon source. All strains were able to grow on these agar media (Fig. 2), while none grew on the control plates with agar as the only carbon source (data not shown). This indicates that all strains had the ability to degrade chitin and mucin.

To learn more about the strains’ antibiotic resistances, the antibiotic susceptibilities of the *Janthinobacterium* strains were tested on LA plates. Most strains showed resistance toward ampicillin (100 µg/ml) and streptomycin (25 µg/ml), however we observed some growth for strain 3.116 when exposed to ampicillin (100 µg/ml) and for pbA and 3.116 when exposed to streptomycin (25 µg/ml). The growth of strain pbA was inhibited when exposed to erythromycin (50 µg/ml), while the growth of the other strains was reduced (Table S10). All the strains were susceptible to kanamycin (50 µg/ml), except for pbB, which showed reduced growth. For tetracycline (10 µg/ml), the only strain which was resistant was 3.108 (Table S10).

### Genomic characterization of the *Janthinobacterium* strains

Since the five strains showed high phenotypic diversity despite having almost identical 16S rRNA gene sequences, we examined their diversity further by genome sequence analysis. The number of reads obtained for the genomes varied between 11.3 and 16.9 million among the strains (Table 2). The GC content ranged from 61.4% to 63.6%, and the number of protein-coding genes varied between 5463 and 5950. The number of contigs varied between 34 and 78, while the length of the longest contig varied from 597 883 to 1 357 233 bp among the strains. By using CheckM, the completeness was found to be >99.2% for all genomes, while the fraction of reads representing contamination was found to be <1.9%.

**Table 2.** General characteristics of *Janthinobacterium* genomes sequenced in this study.

Strain	3.109	3.116	pbA	pbB	3.108
Number of reads	15 354 202	16 913 288	12 555 640	17 208 986	11 274 908
Size (bp)	6 036 801	6 401 392	6 248 925	6 422 512	6 757 357
GC content (%)	61.36	62.19	62.56	63.57	62.53
number of contigs	78	67	47	34	59
Longest contig	636 710	763 058	630 822	1 357 233	597 883
Shortest contig	523	551	631	569	508
N50	200 635	361 185	282 632	386 418	341 651
L50	9	6	7	4	8
Number of predicted genes	5529	5680	5575	5609	6020
Number of protein-coding genes (PROKKA)	5463	5611	5504	5540	5950
Number of genes with nonhypothetical function	2815	2970	2966	2956	3048
Completeness (CheckM, in %)	99.55	99.75	99.25	99.45	99.37
Contamination (CheckM, in %)	1.38	1.28	1.85	1.87	1.64
BGC fraction of genome (%)	3.12	3.15	3.69	4.75	3.45

#### Sequence similarity and phylogenetics of the Janthinobacterium genomes

**Figure 3.**
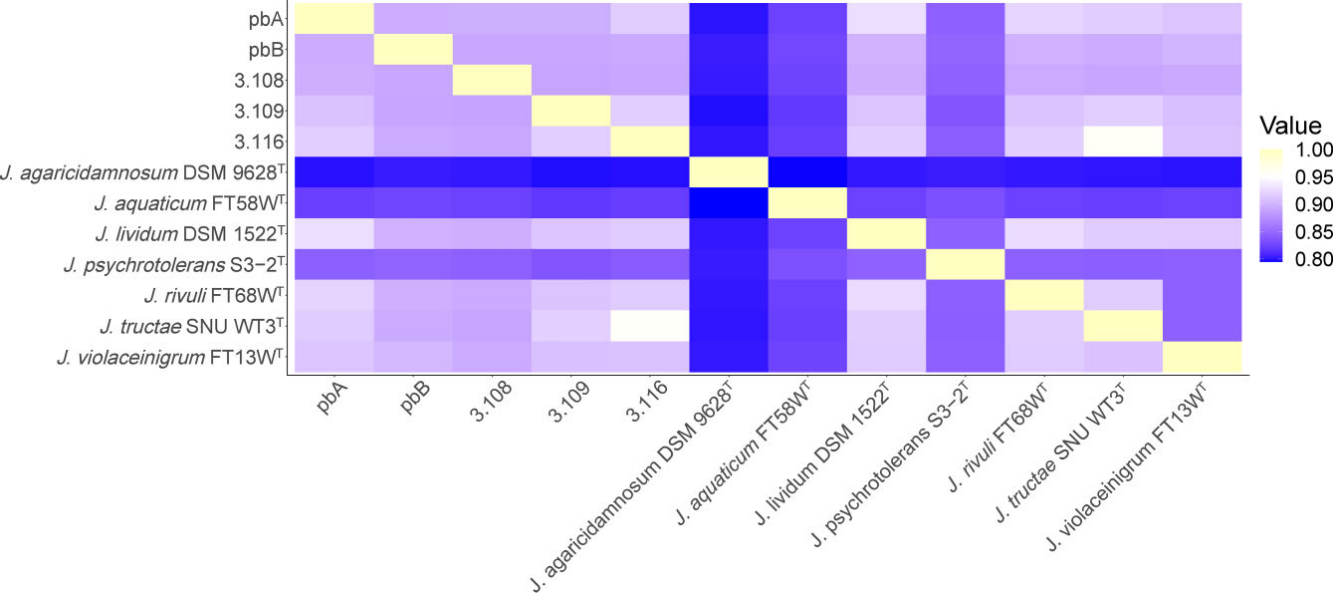
Heatmap showing the sequence similarities based on ANI analyses using FastANI (Jain et al. 2018) between genomes of the five *Janthinobacterium* strains reported in this study and the seven *Janthinobacterium* type strains; *J. tructae* SNU WT3^T^, *J. lividum* DSM 1522^T^, *J. rivuli* FT68WT, *J. violaceinigrum* FT13W^T^, *J. psychrotolerans* S3-2^T^, *J. aquaticum* FT58W^T^, and *J. agaricidamnosum* DSM 9628^T^. The transition from blue to white (at 0.95) marks the threshold for species demarcation. Shades of yellow represent genomes assumed to be the same species.

In contrast to the highly similar 16S rRNA gene sequences, the FastANI analysis indicated that the five strains represented different species with ANI values below 92.8% for all comparisons (Fig. 3), while the species threshold is set to 95% (Konstantinidis and Tiedje 2005, Goris et al. 2007). This was confirmed by isDDH analysis, which resulted in values below the species threshold (70%) for all five strains (Fig. S4). Next, we examined whether the strains belonged to any of the seven *Janthinobacterium* species that have been described by a type strain in the TYGS database (*J. tructae* SNU WT3^T^, *J. lividum* DSM 1522^T^, *J. rivuli* FT68W^T^, *J. violaceinigrum* FT13W^T^, *J. psychrotolerans* S3-2^T^, *J. aquaticum* FT58W^T^, and *J. agaricidamnosum* DSM 9628^T^). The ANI analysis suggested that none of the strains should be assigned to any of these species, except for strain 3.116, which was suggested to belong to the same species as *J. tructae SNU WT3*^T^ (ANI value >95.5%) (Fig 3a). On the other hand, the isDDH analysis indicated that neither 3.116 nor any of the other strains belonged to a previously described *Janthinobacterium* species (Fig. S4). A rMLST analysis using pubMLST identified *J. rivuli* as the genome with highest similarity to the pbA genome (85% support), while the genome of strain 3.116 was classified as a *J. tructae* (82% support) (Jolley et al. 2018). All the remaining strains had lower support (<66%) for their closest relative type strains (Table S4).

**Figure 4.**
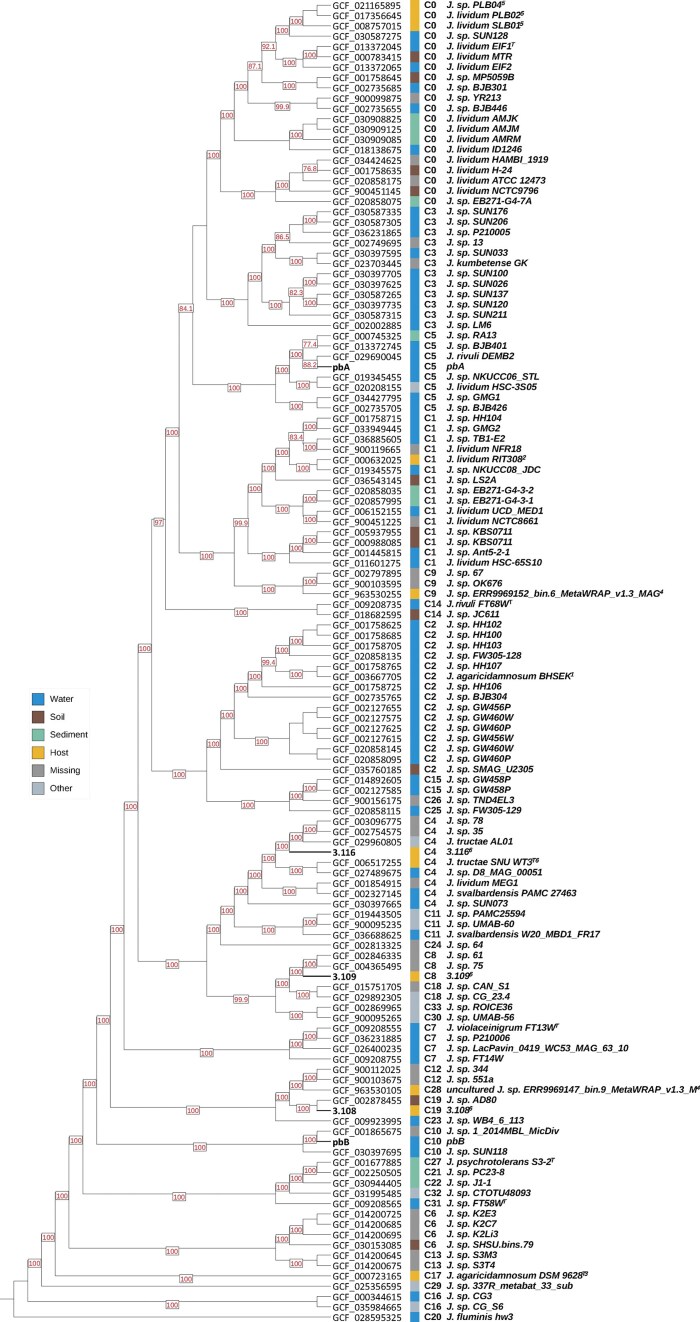
Phylogenetic maximum-likelihood tree constructed from 532 orthologous core genes for all currently available (16 April 2024) *Janthinobacterium* genomes at NCBI using the Q.plant+F+I+G4 model. *J. fluminis* hw3 was used as outgroup as this was the most distant relative (Jain et al. 2018). The colored bars indicate the source environment for each genome, and the numbers following the “C” at the right side of the colored bars represent species demarcation based on FastANI (Jain et al. 2018). The NCBI accession numbers are reported at the tip of the branches. The type strains are marked by the letter T in superscript. Numbers associated with the strain ID refer to the more specific isolation source for some strains originating from water or host; 1. Recirculating aquaculture system, 2. Plant, 3. Fungi, 4. Human, 5. Sponge, and 6. Fish. Bootstrap values above 75 are reported at the nods.

We performed a phylogenetic analysis to further investigate the evolutionary relationships between the genomes of the strains and previously reported *Janthinobacterium* genomes. The analysis was based on 1495 core genes for all currently available *Janthinobacterium* genomes in the NCBI database (129 genomes) and represented genomes originating from various environments (aquatic, soil, sediments, and animal, plant, and fungal hosts). The resulting tree showed that the five strains were not closely related to each other, nor any of the *Janthinobacterium* type strains, except for strain 3.116, which was grouped together with *J. tructae* SNU WT3^T^ (Fig. 4). Generally, there was no obvious relationship between the source environments of the genomes and their relatedness. Still, strains 3.108 and 3.116 had host-associated bacterial genomes among their closest relatives, while pbA and pbB had strains originating from aquatic environments as their closest relatives (Fig. 4).

#### Functional potential of the genomes

**Figure 5.**
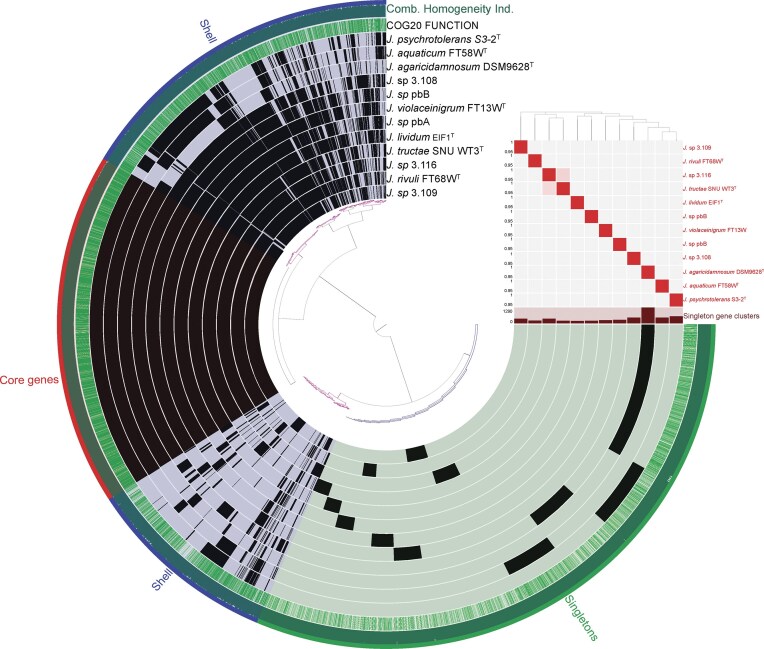
The pangenome of the five *Janthinobacterium* strains reported in this study (pbA, pbB, 3.108, 3.109, and 3.116) and the seven *Janthinobacterium* type strains, (*J. tructae* SNU WT3^T^, *J. lividum* EIF1^T^, *J. rivuli* FT68W^T^, *J. violaceinigrum* FT13W^T^, *J. psychrotolerans* S3-2^T^, *J. aquaticum* FT58W^T^, and *J. agaricidamnosum* DSM 9628^T^) was constructed with the Anvi’o pipeline (Eren et al. 2021). The pangenome was binned into Core genes (*n* = 2906), shell genes (*n* = 25 857), and singletons (*n* = 5094). Several of the genomes were sequenced only to the contig level. This could potentially affect the accuracy of the anayses as incomplete genomes could alter the proportions of core genes, singletons and shell genes.

**Figure 6.**
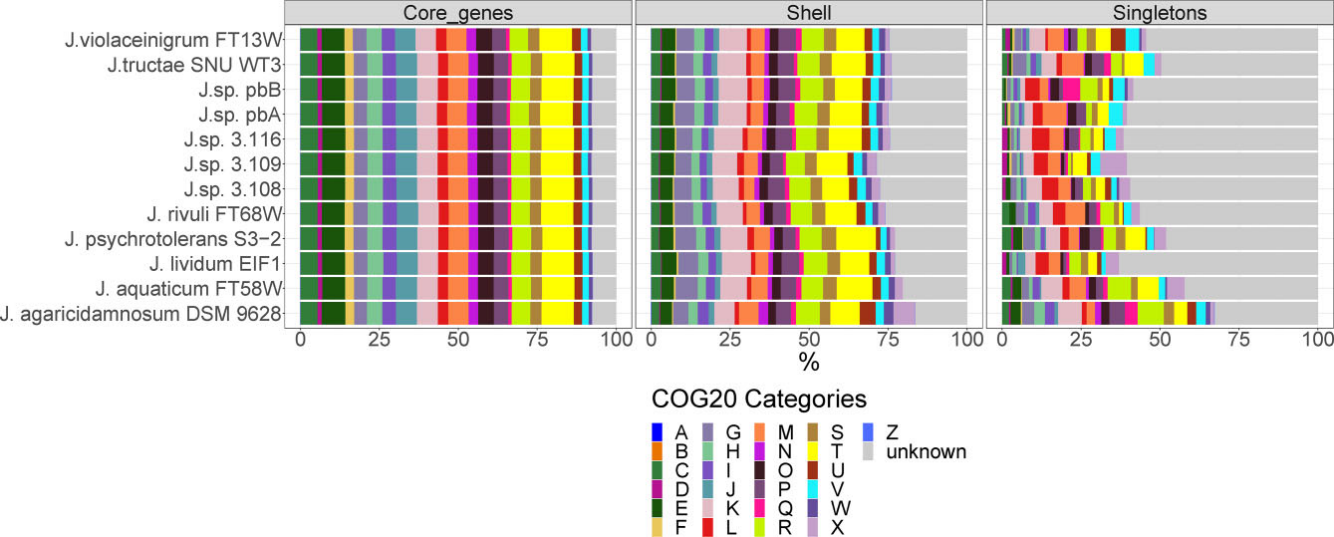
Overview of the functional gene categories associated with the bins from the pangenome for each of the strains. The COG20 categories represent the following categories of gene functions: (A) RNA processing and modification, (B) chromatin structure and dynamics, (C) energy production and conversion, (D) cell cycle control, cell division, and chromosome partitioning, (E) amino acid transport and metabolism, (F) nucleotide transport and metabolism, (G) carbohydrate transport and metabolism, (H) coenzyme transport and metabolism, (I) lipid transport and metabolism, (J) translation, ribosomal structure, and biogenesis, (K) transcription, (L) replication, recombination, and repair, (M) cell wall/membrane/envelope biogenesis, (N) cell motility, (O) posttranslational modification, protein turnover, and chaperones, (P) inorganic ion transport and metabolism, (Q) secondary metabolites biosynthesis, transport, and catabolism, (R) general function prediction only, (S) function unknown, (T) signal transduction mechanisms, (U) intracellular trafficking, secretion, and vesicular transport, (V) defense mechanisms, (W) extracellular structures, (X) mobilome: prophages, transposons, (Y) nuclear structure, and (Z) cytoskeleton.

A pangenomic analysis was conducted to further examine the similarities and differences of the *Janthinobacterium* genomes. The pangenome of the five *Janthinobacterium* strains and the seven *Janthinobacterium* type strains was composed of a total of 65 823 genes (Fig. 5), while the core genomes encompassed of a total of 2906 genes, of which 2491 were single-copy genes. Singletons, i.e. genes that were observed only in one of the genomes, comprised 5094 genes, while the remaining 25 857 genes were assigned to the shell pangenome, i.e. genes that are observed in two or more genomes, but not all (Fig. 5). The distribution of core genes into the COG20 functional categories were highly similar among the strains (Fig. 6). The most abundant functional categories for the core genes were “Signal transduction mechanisms” (10.3 ± 0.1%), “Amino acid transport and metabolism” (7.3 ± 0.1%), and genes with unknown function (7.6 ± 0.1%; Fig. 6). The functional assessment of the singletons and shell genes to the COG20 categories showed more variation among the genomes, in particular this applies to the number of genes assigned to the functional categories “Secondary metabolites biosynthesis,” “Transport and catabolism,” “Defence mechanisms,” “Cytoskeleton,” and genes of unknown function (Fig. 6). The greatest difference to the core genes was found for the category “Mobilome: prophages, transposons,” with an average abundance of 0.08 ± 0.02%, 2.5 ± 1.5%, and 3.2 ± 2.1% for the Core genes, shell genes and singletons, respectively (Fig. 6). Furthermore, some of the functional categories that were represented only by singletons were found only in some of the genomes. For example, “Intracellular trafficking, secretion, and vesicular transport” was found only in 9 of the 12 genomes. At the same time, it represented as much as 4.7% of the singletons for *J. rivuli* FT13W^T^.

We investigated the functional potential of the genomes of the five strains further by using NCBI BLAST to search for genes previously shown to be characteristic of members of the *Janthinobacterium* genus (Table S5). By using JQS genes from *Janthinobacterium* sp. HH01 (GCA_000335815.1) as query sequences, we found that all five strains contained the putative genes (*jqsA, jqsR*, and *jqsS*) for this QS system (Table S5). To examine the potential presence of AHL QS systems, we used the genes encoding homoserine lactone synthase (*luxI*/*lasI*) and the transcriptional regulator (*luxR*) of the *J. lividum* type strain (accession number: GCA_001758 635) as queries. Putative *luxI* and *luxR* genes were identified in the genomes of strain 3.108, pbA, and pbB, but with relatively low sequence identities (Table S5). These genes had been annotated as homoserine lactone synthase and transcriptional activator proteins. Next, we examined the genetic potential of the strains for carbon fixation, a function that has been associated with capnophilic properties of bacteria and searched for genes encoding phosphoenolpyruvate carboxylase and those encoding enzymes in the glyoxylate cycle (malate dehydrogenase, citrate synthase, malate synthase, isocitrate lyase, and aconitase). These putative genes were found for all five strains (Table S5). Since all the strains were able to utilize mucin and chitin as growth substrates, we further examined the presence of genes known to be involved in the degradation of chitin and mucin by screening for carbohydrate-active enzymes using dbCAN. All five genomes putatively encoded the chitinases glycoside hydrolase GH18 and GH19 (Table 3). However, the glycoside hydrolases that are assumed to be involved in mucin degradation pathways were lacking (Table S6). Thus, the genetic potential for mucin degradation was not identified.

**Table 3.** The number of chitinase glycoside hydrolases GH18 and in the *Janthinobacterium* genomes of strains pbA, pbB, 3.108, 3.109, and 3.116 determined by dbCAN (Zheng et al. 2023).

Strain	GH18	GH19
pbA	4	2
pbB	5	3
3.108	3	3
3.109	3	2

The genomes of the five strains were investigated for their potential for antibiotic resistance by using ARTS2, CARD, and ResFinder. Putative genes encoding tetracycline resistance were found for the strains pbA and 3.108 (Tables  S7 and S8). Furthermore, putative genes encoding efflux pumps associated with fluoroquinolone, tetracycline antibiotic resistance, and an efflux pump involved in resistance toward disinfecting agents and antiseptics were identified for all five strains (Table S8). The genomes of the strains pbA, pbB, and 3.116 also contained genes encoding resistance against phosphonic acid antibiotics (Table S8).

#### Secondary metabolite gene clusters

**Figure 7.**
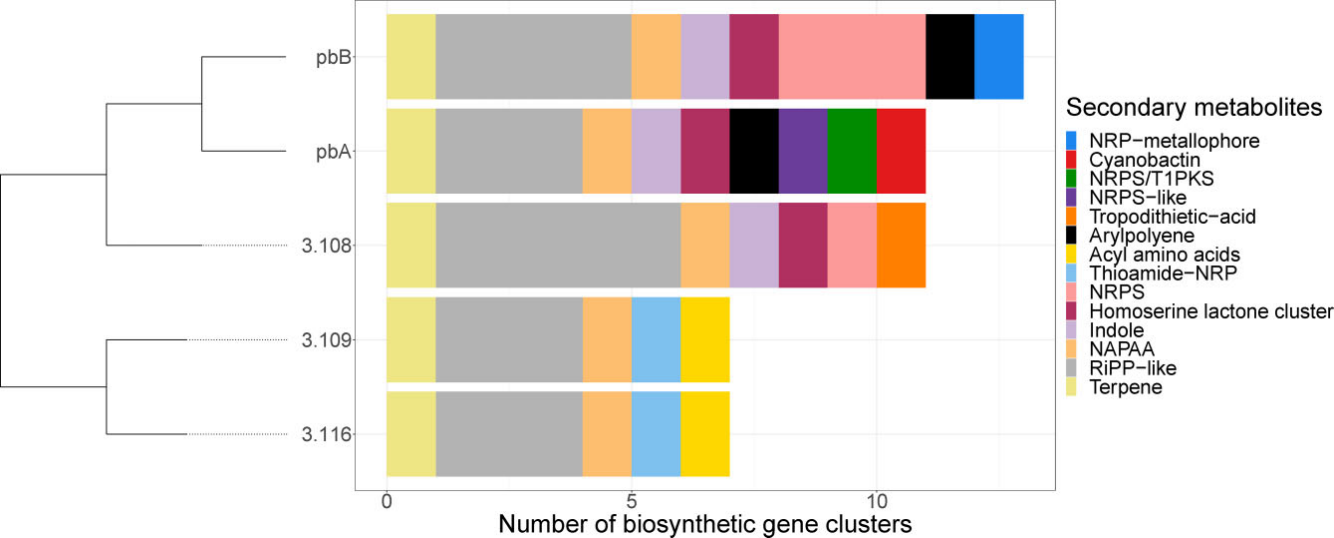
Secondary metabolite gene clusters predicted by AntiSMASH v7.1.0 (Blin et al. 2023) for the five *Janthinobacterium* genomes reported in this study. The maximum-likelihood phylogenetic tree is based on single-copy core genes. The length of the horizontal bars indicates the number of BGCs within each class of secondary metabolites. As the genome sequences were not closed, the number of BGCs could potentially be underestimated.

As members of the genus *Janthinobacterium* are known for producing secondary metabolites, we examined the genetic potential of the strains for the biosynthesis of secondary metabolites. We estimated that each of the five *Janthinobacterium* genomes harboured between 7 and 13 predicted secondary metabolite BGCs (Fig. 7), with the highest numbers found for pbA, 3.108 and pbB (11, 11, and 13, respectively). This corresponded to between 3.1% and 4.8% of their genome sequences, with the highest fraction found for strain pbB (Table 2). To assure that the BCGs were not overestimated due to the lack of complete genome sequences, we inspected the placement BCGs on each contig to check for potential duplicates. This revealed that one of the strain pbB have a NRPS at the end of contig two, and at the beginning of contig 3. However, based on alignments this seems to be two different NRPSs. The remaining strains had no suspicious BCGs. The number of RiPP-like (ribosomally synthesized and posttranslationally modified peptides) BGCs varied from three to five between the strains (Fig. 7). The genomes of the strains all contained one non-alpha poly-amino acids (NAPAA) gene cluster, which putatively encodes d-alanine—d-alanyl carrier protein ligase. Furthermore, all the genomes contained one terpene gene cluster consisting of three genes encoding hydroxysqualene synthase, 15-cis-phytoene synthase, and hydroxysqualene dehydroxylase. Putative gene clusters encoding indoles, in this case, violacein, were found for the strains pbA, pbB, and 3.108, the same strains that were found to form purple colonies under some conditions. Furthermore, as described above, the same three strains were found to possess homoserine lactone clusters (AHL-QS system). Putative genes encoding acyl amino acids and thioamide-containing non-ribosomal proteins (thioamide-NRP) were found only for 3.109 and 3.116 (Fig. 7). Additionally, genes potentially involved in the biosynthesis of tropodithietic acid (TDA) were identified in the genome of strain 3.108. To investigate whether these genes could be present in the genomes of the other strains, the TDA biosynthesis operon, *tdaABCDE*, in *Phaeobacter inhibens* strain P10 (accession number: GCA_002888685.1) was queried against their genomes using protein BLAST. The genome of 3.108 remained the only genome carrying similar genes with a % identity of 49.3%–69.6% and E-values of 4.68e^−71^–4.46e^−131^; however, lacking a match for *tdaC*. Any hits in the other genomes had higher E-values (>5.99e^−26^) and low % identity (<42.1%), suggesting that these genes are lacking. Furthermore, the organization of the genes in the TDA operon of 3.108 differed from that in *P. inhibens* (Fig. S5). The genetic potential for biosynthesizing non-ribosomal peptide metallophores (NRP-metallophore) was found only for the strain pbB (Fig. 7).

#### Phages and virulence factors

The number and identity of prophage sequences varied between the genomes of the five *Janthinobacterium* strains (Table S11). A total of six intact prophage sequence regions were identified, with sequence lengths between 24.2 and 50.5 kb. Three of the intact prophages were found in strain 3.108, where two of them were annotated as *Burkholderia* phages (KS9 and Bcep176), and the last was annotated as a *Pseudomonas* phage (YMC11/02/R656) (Table S11). The strains pbA, pbB and 3.109 all contained one intact phage sequence each, all annotated as a *Burkholderia* phage. The strains pbA and pbB share the same phage sequence for the *Burkholderia* phage KS14, while for 3.109, the intact phage sequence was found to represent a *Burkholderia* phage phi1026b (Table S11). No intact phage regions were identified in the genome of 3.116 (Table S11).

All genomes carried genes encoding virulence factors, mostly flagella or type IV pili (Table S12). Flagella-encoding genes were identified in the genomes of all strains, while putative genes for pili were found in all, except for pbA. This, together with several chemotaxis genes found in all genomes (Table S13), suggests a potential for motility. pbB possessed putative genes encoding several additional virulence factors: the XCP secretion system (a type II secretion system), isocitrate lyase, an enzyme of the glyoxylate cycle, and the toxin adenylate cyclase. By using NCBI BLAST, Isocitrate lyase was identified also in the genomes of the remaining strains. The strain 3.108 possessed the genes putatively encoding the virulence factor KatA (Table S12).

### Colonization of Atlantic salmon yolk sac fry by the *Janthinobacterium* strains

**Figure 8.**
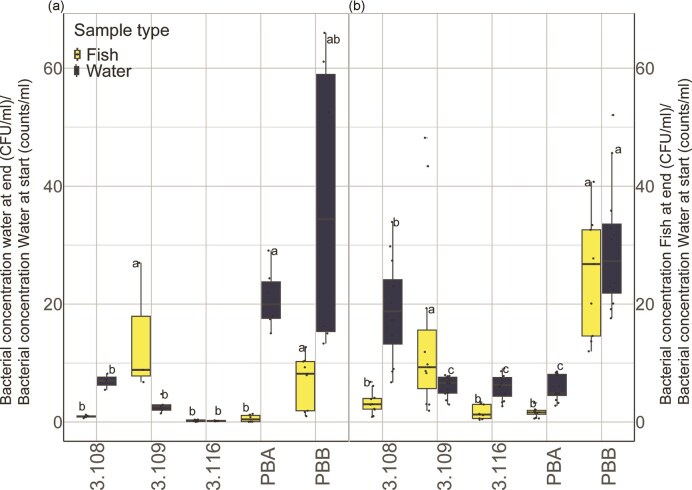
The bacterial concentrations in the water and of the fish at the end of the experiments given as ratios to the concentrations in the water at the start of the experiments. The number of colony-forming units per ml (CFU/ml) was counted after cultivation of dilutions of homogenized yolk sac fry or water samples on TSA agar plates. Germ-free yolk sac fry was cultivated in SGM before and after the addition of the different *Janthinobacterium* strains. The concentration of bacteria in the water at the start of the experiment was determined using flow cytometry, and the bacterial densities (based on CFU) in water and fish samples were normalized to the density in the water directly after adding the strains. The boxes represent median ± interquartile range, while the whiskers represent 1.5*IQR. For (a) Experiment 1, lasting 22 days postexposure, one flask was used for each strain, three fish from each flask were used and two replicate water samples were used from each flask. For (b) Experiment 2, lasting 26 days postexposure, four replicate flasks were used with one fish and one water sample from each flask. The letters represent significant differences based on ANOVA tests between the samples from each experiment and each sample type (*P* > .05). For each sample, three replicate agar plates were counted. Due to an unexpectedly high number of CFUs for some of the agar plates in the CFU analyses, one biological replicate fish sample (three agar plates) was disregarded for each of strains 3.108, 3.109, and pba in Experiment 1. For experiment 2, one fish sample (three agar plates) was disregarded for 3.116, while and one replicate water sample (three agar plates) was disregarded for the water samples of 3.108 in experiment 1.

Three of the *Janthinobacterium* strains originated from salmon skin samples (3.108, 3.109, and 3.116), while pbA and pbB were isolated from rearing water in cultivation flasks with yolk sac fry. To investigate whether the source environment for the strains (rearing water or salmon skin) was related to their potential to colonize salmon fry, we exposed germ-free eggs close to hatching to each of the five strains through the rearing water in two separate experiments (Exp.1 and Exp.2). When the eggs were hatched, the yolk-sac fry were exposed to the bacteria on the egg surface and in the rearing water. We aimed at adding the strains to a concentration of 10^5^ cells/ml, and flow cytometry analysis showed that the average concentration of bacterial cells in the rearing water at the start of the experiment was 2.7 × 10^4^ ± 1.5 × 10^3^ cells/ml (Table S14). During the experiments, no fish mortality was recorded for experiment 1, while one out of 246 fish died in experiment 2. At the end of the experiments, 22 and 26 days after exposure to bacteria, for Exp.1 and Exp.2, respectively, most of the strains were found to be present in the rearing water in similar or higher concentrations than at the start of the experiment (between 8.5 × 10^4^ and 8.0 × 10^5^ cells/ml; Table S14). The results varied somewhat between the two experiments, but there was a considerable increase in density of 3.108 and pbB in the rearing water in both experiments (Fig. 8), indicating bacterial growth in the rearing water. This was also the case for strain pbA in Exp.1. For strains 3.116 and 3.109, the bacterial growth in the water appeared to be considerably lower, and in Exp.1, the concentration of 3.116 did not increase, thus indicating a lack of bacterial growth in the rearing water. To estimate the strains’ ability to colonize salmon yolk sac fry, we used homogenized larvae in CFU analysis. The average number of CFUs per ml of larval homogenate varied extensively among the strains; from 8.6 × 10^2^ ± 2.2 × 10^2^ CFU/ml to 1.5 × 10^6^ ± 7.0 × 10^4^ CFU/ml for Exp.1 and Exp.2, respectively (Table S14), but also between replicate samples. These results indicate that all five strains colonized the salmon fry. Since the bacterial concentration in the water at the start of the experiment varied among the strains, we further normalized the bacterial densities of the larval homogenates to the bacterial concentration of the rearing water at the start of the experiment (Fig. 8). The ratio between the bacterial concentrations of larval homogenate and of the rearing water at the start of experiments was found to be on average 14.2 ± 6.4 and 15.2 ± 4.8, for Exp.1 and Exp.2, respectively, for strain 3.109. The same ratio was found to be on average 7.0 ± 1.5 and 24.6 ± 3.4 for Exp.1 and Exp.2, respectively, for strain pbB. These ratios were significantly higher than for the remaining strains (ANOVA, *P* > .05, Fig. 8).

## Discussion

In this study, through the phenotypic- and genome-based approaches, we demonstrated a high phenotypic, genomic, and functional diversity of *Janthinobacterium* associated with the skin and the rearing water of Atlantic salmon fry. We have previously observed high relative abundances of this genus in the microbiota associated with yolk sac fry of Atlantic salmon (Fiedler et al. 2023). However, that study, as most studies involving the characterization of microbiomes, was based on the sequencing of 16S rRNA amplicons. Due to the highly similar 16S rRNA gene sequences of the five strains and seven type strains (>98%, Table S15), such studies would not reveal the high diversity of this genus. If based on 16S rRNA gene sequences, these 12 strains would be classified to the same species, while their genome sequences suggested that they belong to 11 distinct species (Table S15). We further compared the evolution of the 16S rRNA gene sequences and protein-coding genomic genes by constructing Maximum Likelihood phylogenetic trees based on both 16S rRNA gene sequences and the core genes of the strains (Fig S6). The branch lengths of the tree based on 1060 core genes were generally around 10 times longer than those in the tree based on the 16S rRNA gene sequences (Fig. S6). Moreover, the average ANI for these 12 strains was 88.4 ± 0.5%, while the average 16S rRNA gene sequence identity for these 12 strains were 99.57 ± 0.04%. These analyses indicate a low evolutionary rate of the 16S rRNA gene in *Janthinobacterium*. This is a phenomenon that has been observed for other bacterial genera, and that has been suggested to be explained by evolutionary diversification through horizontal gene transfer (Bartoš et al. 2024).

Despite the highly similar 16S rRNA gene sequences of the five strains, the strains differed phenotypically. A striking difference was the color. Two of the strains, pbA and pbB, formed purple colonies on most of the tested agar media. Absorption maximums for the purple pigment were similar to that previously reported for violacein extracted from *C. violaceum* (Rettori and Durán 1998). Strain 3.108 formed purple colonies under specific growth conditions, e.g. LA plates with reduced agar concentrations. In accordance with the observed phenotypes, only 3.108, pbA, and pbB carried genes encoding the purple pigment violacein. A purple phenotype and violacein production has often been considered to be characteristic of the genus *Janthinobacterium* (Pantanella et al. 2007, Wu et al. 2021). However, *Janthinobacterium* strains that are not capable of producing violacein, or that are lacking the purple phenotype, have been described previously (Kumar et al. 2018, Wu et al. 2021). In this study, the strains originating from water (pBA and pBB) had a deep purple phenotype, while the three strains originating from salmon skin, usually lacked this characteristic. Interestingly, the only other *Janthinobacterium* isolated from fish, *J. tructae* SNU WT3^T^, also lacked the genetic potential to produce violacein (Jung et al. 2021). Since only a few fish-associated *Janthinobacterium* strains have been characterized so far, it remains to be seen whether this phenotype is characteristic to strains colonizing mucosal surfaces of fish.

We found that all five strains could utilize chitin as a carbon source, and found that they possessed genes encoding chitinases belonging to both the GH18 and GH19 families, explaining their ability to degrade chitin (Kawase et al. 2006, Berlemont and Martiny 2015, Han et al. 2016). The ability to degrade chitin has previously been associated with antifungal properties in *Janthinobacterium* (Haack et al. 2016). Chitinases of the GH19 family are commonly found in plants. However, some bacteria belonging to the genera *Vibrio* (Honda et al. 2008)*, Streptomyces* (Ohno et al. 1996, Watanabe et al. 1999, Itoh et al. 2002, Kawase et al. 2006), and *Pseudoalteromonas* (Paulsen et al. 2019) have also been found to possess these genes. Furthermore, these chitinases have been suggested to be involved in antifungal properties in *Streptomycetes* (Watanabe et al. 1999, Kawase et al. 2006). All the strains also grew on an agar medium with mucin as the only carbon source. This indicates that the strains have the potential to utilize mucus for growth, for example in the skin mucus of fish. However, we did not identify the glycoside hydrolases previously found to be involved in mucin degradation (Glover et al. 2022, Raba and Luis 2023) in any of the genomes, suggesting an alternative way of degrading these compounds might be occurring in this case.

We sequenced the genomes by using an Illumina Novaseq 6000 instrument, which resulted in a relatively large number of contigs per genome, i.e. the genomes were not complete. The FastANI, isDDH, and PubMLST analyses have been described to be relatively robust towards incomplete genomes (Jain et al. 2018, Jolley et al. 2018, Meier-Kolthoff and Göker 2019). Further, the phylogenetic tree is based on core genes present in all the analysed genomes, thus it should not be severely affected by the high number of contigs. The analyses of secondary metabolites, pangenome and regions representing phages and prophages, on the other hand, might have been inaccurate due to the incompleteness of the genome sequences. The results from the pangenome analyses might be affected by contig-level genomes. Incomplete genomes could result in wrong estimation of the number of genes, as gene fragments at the end of contigs could be annotated and counted on several different contigs (Salzberg 2019, Ceres et al. 2022), and some genes could be lacking. These factors can result in a skewed fraction of core genes, and an overestimation of unique genes. The number of phage and prophage sequences is often underestimated as mobile genetic elements are usually underrepresented for genomes that are not completely determined, as these typically contain repeats, and thus are difficult to assemble correctly (Page et al. 2018, Zhang et al. 2023). Long-read sequencing, i.e. using the nanopore technology, would most likely have resulted in a closed genome, future analyses of these Janthinobacterium isolates should therefore be confirmed by long-read sequencing.

Phylogenetic analyses based on 532 core genes showed that none of the five strains were closely related and that only strain 3.116 was closely related to any of the *Janthinobacterium* type strains, namely *J. tructae* SNU WT3^T^. ANI analysis suggested that 3.116 belongs to this species (ANI value >95.5%), but not the isDDH and pubMLST analyses. Interestingly, this was the only genome representing a *Janthinobacterium* strain previously isolated from fish, more specifically from the kidney of a diseased rainbow trout *Oncorhynchus mykiss* (Jung et al. 2021). The phylogenetic analysis further showed that most of the available *Janthinobacterium* genomes are not closely related to any of the type strains within this genus, and thus, a number of *Janthinobacterium* species remains to be described.

We performed a pangenome analysis to determine the core genome for the five strains and the seven type-strains for the *Janthinobacterium* genus. The core genome comprised 53% of the genes of the 12 genomes (2906 genes). However, for the phylogenetic analysis (129 genomes) only around 19% of the genes were part of the core genome. Thus, as the core genome decreases substantially with the addition of more strains, it seems to be an open pangenome. A similar pattern has previously been found in several other studies of *Janthinobacterium* pangenomes (Haack et al. 2016, Wu et al. 2021).

Interestingly, this genus appears to be abundant in both the human (Grice et al. 2008), fish (Fiedler et al. 2023; this study), and amphibians (Brucker et al. 2008, Becker et al. 2009, Harris et al. 2009, Bresciano et al. 2015) skin microbiota, although these skin tissues are highly dissimilar. Members of the *Janthinobacterium* genus are known to protect amphibians against fungal infections (Brucker et al. 2008, Becker et al. 2009, Harris et al. 2009). Whether they have a similar function on salmonid skin remains to be studied. A previous study has shown that the growth of the strain *J. lividum* MTR was promoted at heightened CO_2_ concentrations (Valdes et al. 2015). This behavior was proposed to be linked to heterotrophic carbon fixation due to the presence of enzymes in the glyoxylate cycle (malate dehydrogenase, citrate synthase, malate synthase, isocitrate lyase, and aconitase), and phosphoenolpyruvate carboxylase (Valdes et al. 2015). Heterotrophic carbon fixation is known to be a significant contributor to CO_2_ flux in the carbon cycle (Braun et al. 2021) and was first described in propionic acid bacteria (Wood and Werkman 1938). Our *Janthinobacterium* strains were found to possess these genes, and thus, they could potentially be capnophilic. Both amphibians (Emílio 1974) and salmon yolk sac fry (Wells and Pinder 1996) respire and excrete CO_2_ through the skin. Thus, heightened CO_2_ concentrations in the skin of the yolk sac fry could be a mechanism for the recruitment of *Janthinobacterium*.

The five *Janthinobacterium* strains were found to have between 3.1% and 4.8% of their genomes dedicated to BGCs, and they possessed the genetic potential to synthesize diverse secondary metabolites, 14 in total. BGCs on the edges of the contigs were manually investigated for potential duplicates. The only strain, which had a BCG class on the edge with more than one annotation was pbB. This strain had one NRPS on the edge of contig 2 and one on the edge of contig 3. However, alignments indicate that this is two distinct NRPSs. For the remaining strains, an underprediction of the number of BGCs is more likely than an overprediction. Previous studies have found the average prokaryote to dedicate 3.7 ± 3.1% of its genomes to BCG (Cimermancic et al. 2014). However, a *Streptomyces* was found to devote 22% of the genome to BGCs (Cimermancic et al. 2014). Of the 14 different BGCs identified, three were shared between all five strains. The number of BGCs is in line with the findings of Wu et al. (2021), who found that eight *Janthinobacterium* strains isolated from groundwater or sediment had between 6 and 18 BGCs, while the *J. lividum*^T^ type strain had 32. Many of the BGCs we identified were the same as the ones identified by Wu et al. (2021), but we also identified BGCs encoding NAPAA. Wu et al. (2021) found genes encoding bacteriocin in the genomes of all their strains, this group has been renamed to RiPP-like, which we found genes for in all our strains. Together, this indicates that members of the *Janthinobacterium* genus have a great potential for production of diverse secondary metabolites. The strains 3.116 and 3.109 encoded the same classes of BGCs, although the phylogenetic analysis indicated they were not closely related. For the 3.108 strain, we identified a gene cluster putatively encoding the antimicrobial agent tropodhietic acid (TDA). TDA, which is encoded by the operon tdaABCDE, has mainly been described for the marine family *Rhodeobacteracea* (Henriksen et al. 2022). However, two recent studies also identified a similar operon in 5 out of a total of 11 *Janthinobacterium* strains examined (Belikov et al. 2021, Wu et al. 2021), but so far, the synthesis of TDA in *Janthinobacterium* remains to be verified.

The JQS system has been reported to be present in most of the *Janthinobacterium* genomes that have been examined (Hornung et al. 2013, Haack et al. 2016, Wu et al. 2021) and was identified for all five strains. Furthermore, *Janthinobacterium* pbA, pbB and 3.108 also possessed genes associated with the AHL QS system. Interestingly, these were the same strains that carried the violacein operon. A previous study has shown that a few strains of *Janthinobacterium* can harbour both JQS and AHL QS systems (Wu et al. 2021). Wu et al. (2021) found that eight of 50 strains both had the AHL and JQS QS systems. These strains were found to be the strongest violacein producers, and Wu et al. (2021) argued that the additional QS system could be associated with the potential to produce violacein. Togheter with our findings this indicate a possible link between violacein production and an additional AHL QS system. Wu et al. (2021) also identified the gene encoding the transcriptional activator related to AHL synthase, *AnoR*, for three of these 50 genomes. We identified this gene in the genomes of pbA and pbB, while 3.108 had a gene annotated as transcriptional activator *LasR*, which is another activator involved in QS (Kiratisin et al. 2002). We only observed light purple colonies for strain 3.108 when grown under certain conditions, even though this strain possesses violacein genes and the genes encoding the AHL QS system.

We found that all the strains possessed genes encoding virulence factors involved in motility, surface adherence, and biofilm formation. Such factors have been found to be important for the ability to colonize hosts, not only for pathogens but also for commensal host-associated bacteria (Ribet and Cossart 2015, Flemming et al. 2016, Powell et al. 2016, Raina et al. 2019, Obeng et al. 2021). The presence of these genes further substantiates that these *Janthinobacterium* strains can have a host-associated lifestyle. The strains pbB and 3.108 had additional virulence factors. For strain 3.108, the putative gene for the virulence factor *KatA* was found. *KatA* is a catalase associated with resistance to H_2_O_2_ stress, which is common in bacteria (Chelikani et al. 2004, Chung et al. 2016), and it is an important virulence factor for *Pseudomonas aeruginosa* (Lee et al. 2005). The strain pbB encoded multiple virulence factors; Adenylate cyclase (*CyaB* gene), Isocitrate lyase, and an XcpT secretion system. In addition to being considered a virulence factor, Isocitrate lyase is one of the enzymes in the glyoxylate cycle and was also found in the genomes of the other four strains. Isocitrate lyase seems to be important for the persistent infection of some pathogens, however, it is mainly associated with their ability to proliferate (Wall et al. 2005). Thus, it could be an important factor in a host-associated lifestyle. The virulence factor *cyaB* is classified as an intracellular toxin that can damage membranes and is an offensive virulence factor (Liu et al. 2022), however, it is also involved in the synthesis of the signaling molecule cyclic AMP (Cha et al. 2010, Patra et al. 2023).

We found that all five strains were able to colonize yolk sac fry, but the colonization success varied among the strains. Strain 3.109 and pbB had the highest potential for colonizing the yolk sac fry. Strain pbB was originally isolated from the rearing water of the yolk sac fry, while 3.108 and 3.109 were isolated from the skin of the fry. Thus, there was no clear correlation between the source material from which the strains were isolated and their ability to colonize water or yolk sac fry. Furthermore, we cannot exclude that the bacteria isolated from the water could originate from the fish, or vice versa, that the bacteria isolated from fish could originate from the water. Although most of the previously described *Janthinobacterium* members have been isolated from water (Fig. 4), the genus has been observed to be abundant members of diverse animal-associated microbiomes such as amphibians (Brucker et al. 2008, Becker et al. 2009, Bresciano et al. 2015), humans (Grice et al. 2008, Gonzalez et al. 2021, Xu et al. 2021), fish, and ticks (Galaviz-Silva et al. 2022). Except for their protective effect against fungal infection on amphibians and plants, little is known about how these bacteria interact with and colonize their hosts. More studies are needed to elucidate their potential impact on vertebrate host health and to understand the importance of their characteristic properties in a host-associated lifestyle, such as capnophilic growth, heterotrophic carbon fixation, QS, and secondary metabolite synthesis.

## Conclusion

Through the comparative phenotypic and genomic analyses in this study, we demonstrate a high phenotypic, genomic, and functional diversity of *Janthinobcterium* in the skin and rearing water of Atlantic salmon fry. Despite having almost identical 16S rRNA gene sequences, the five strains had distinct phenotypes, and genome sequencing suggested that they represented different species. This shows that amplicon sequencing of 16S rRNA gene sequences is not suited for assessing the diversity of this genus. Only one of the strains appeared to be related to a previously described species in this genus. All the strains were able to utilize mucin and chitin as carbon sources, and three were found to have the capacity to produce the pigment violacein. Their genomes encoded a diverse range of other secondary metabolites, but the repertoire varied among the strains. While the genomes of all the strains included gen clusters putatively encoding one terpene, one putative NAPAA, and several RiPP-like factors, one included an operon with genes required for the synthesis of TDA, a secondary metabolite that previously mainly has been described for members of the marine genus *Phaeobacter*. Furthermore, all genomes encompassed genes for the JQS system, and three of the strains had genes encoding an additional AHL QS system. The same three strains also had the capacity to produce violacein, further indicating a possible link between the additional AHL QS system, and violacein production. All the strains were resistant to ampicillin (100 µg/ml) and streptomycin (25 µg/ml), and genes involved in antibiotic resistance were found for all strains. All strains carried genes putatively encoding enzymes of the glyoxylate cycle, a pathway that has been associated with heterotrophic carbon fixation and capnophilic properties. We found several indications that these strains have a host-associated lifestyle. First, three of the strains were isolated from the skin of Atlantic salmon fry. Second, they were able to grow on mucin as the only carbon source. Third, their genomes contained genes associated with properties typically associated with a host-associated lifestyle, such as motility and chemotaxis. Finally, all were able to colonize Atlantic salmon yolk sac fry, although at varying success. This study demonstrated a high diversity of *Janthinobacterium* associated with yolk sac fry of Atlantic salmon. Further studies are needed to understand how they interact with their hosts and to clarify their potential influence on host development and health.

## Supplementary Material

xtaf015_Supplemental_Files

## Data Availability

The flow cytometry data (.fcs files) are available at https://doi.org/10.6084/m9.figshare.28211000.v1. The *Janthinobacterium* genomes are available at NCBI under the BioProject: PRJNA962300, with the following accession numbers: Strain 3.116 (JASAUM000000000), strain 3.109 (JASAUN000000000), strain3.108 (JASAUO000000000), strain pbB (JASAUP000000000), and strain pbA (JASAUQ000000000). The scripts used for the bioinformatics are publicly available through github https://github.com/eidelo/Genomic-diversity-Janthinobacterium.
