# Supplementary material for: Large genomic, functional, and phenotypical diversity of Janthinobacterium associated with Atlantic salmon fry
Source: FEMS Microbes. 2025 Oct 29;6:xtaf015. doi: 10.1093/femsmc/xtaf015 (PMC12618000; doi:10.1093/femsmc/xtaf015)
Supplement: xtaf015_Supplemental_Files [file xtaf015_supplemental_files.zip › FEMSMC-2025-006.R2 one sentence summary.docx]

Five strains of the genus Janthinobacterium isolated from systems with Atlantic salmon fry exhibit large genetic and phenotypic diversity and appear to have a host-associated lifestyle.
