# Supplementary material for: Large genomic, functional, and phenotypical diversity of Janthinobacterium associated with Atlantic salmon fry
Source: FEMS Microbes. 2025 Oct 29;6:xtaf015. doi: 10.1093/femsmc/xtaf015 (PMC12618000; doi:10.1093/femsmc/xtaf015)
Supplement: xtaf015_Supplemental_Files [file xtaf015_supplemental_files.zip › Supplementary_figures.docx]

Supplementary figures and tables for:

**Large genomic, functional, and phenotypical diversity of Janthinobacterium associated with Atlantic salmon fry**

Eirik Degre Lorentsen^1^, Eva C. Sonnenschein^2^, Alexander W. Fiedler^1,3^,Ingrid Bakke^1^

1 Norwegian University of Science and Technology, Trondheim, Norway

2 Swansea University, Swansea, Wales, United Kingdom

3 Enova, Trondheim, Norway


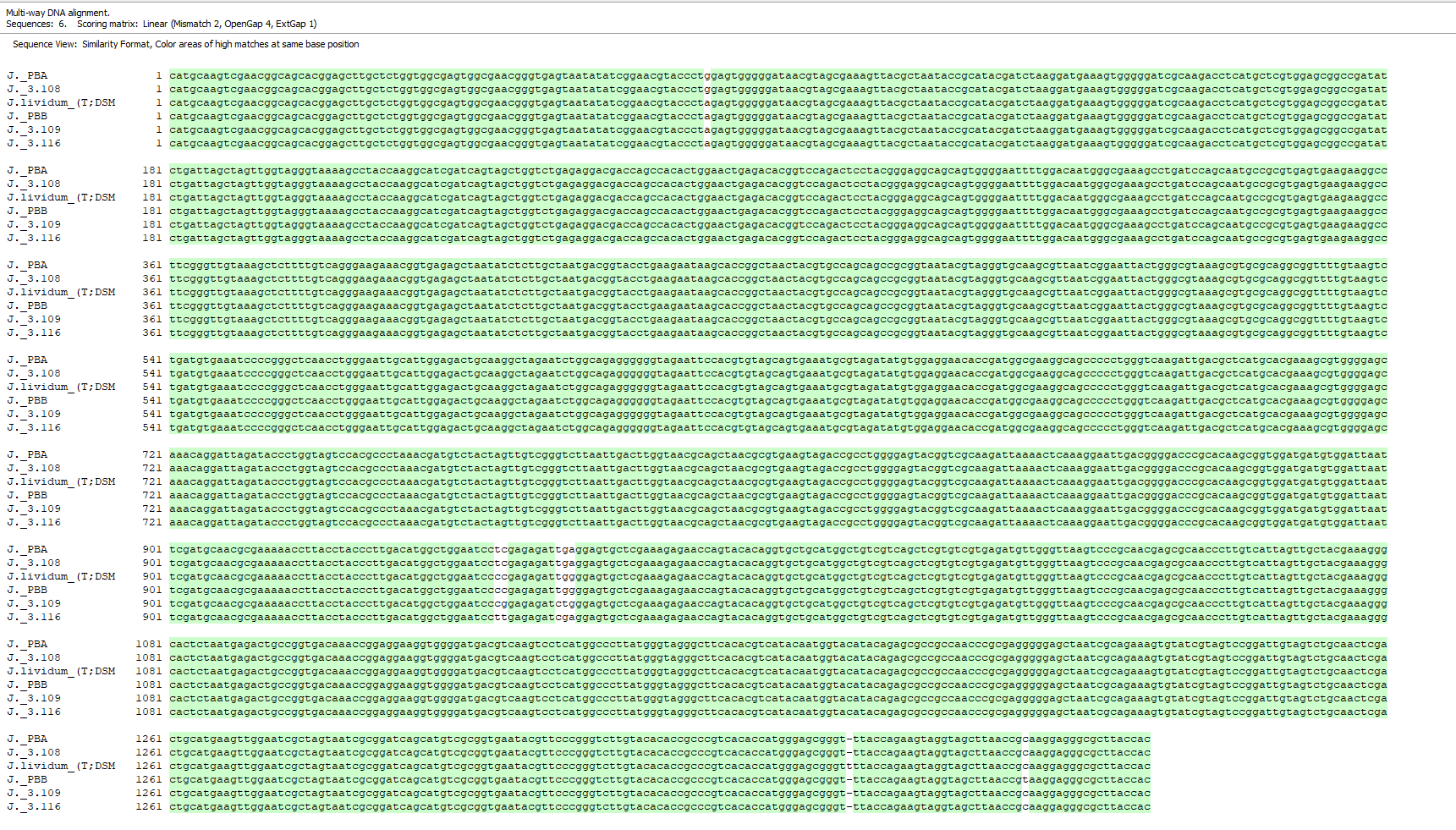


Figure S1: Alignment of 16s rRNA gene sequences for the five *Janthinobacterium* strains, 3.108, 3.109, 3.116, pbA and pbB with the *Janthinobacterium lividum* type strain generated by using multi-way alignment in Clone Manager software (version 9.51). The gene sequences resulted from sanger sequencing of PCR products generated by the primer pair EUB8. and 1492.R (Weisburg *et al.* 1991).


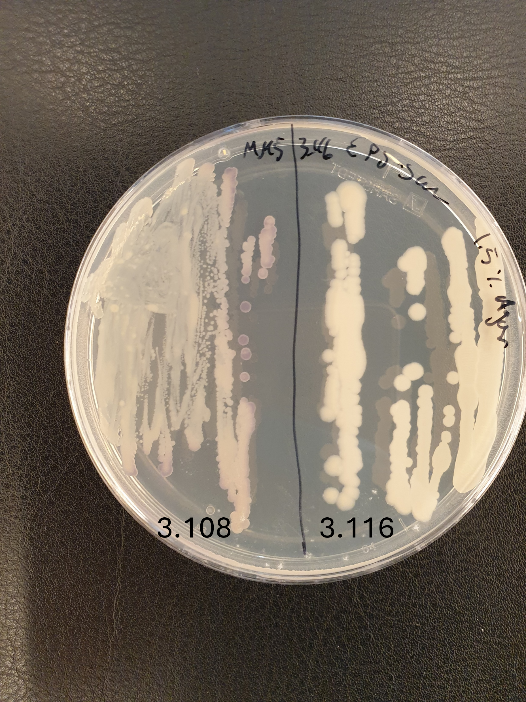


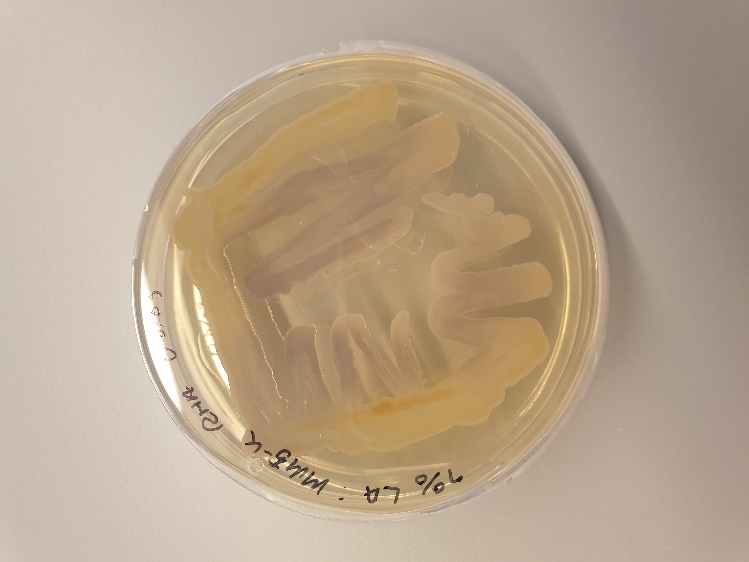


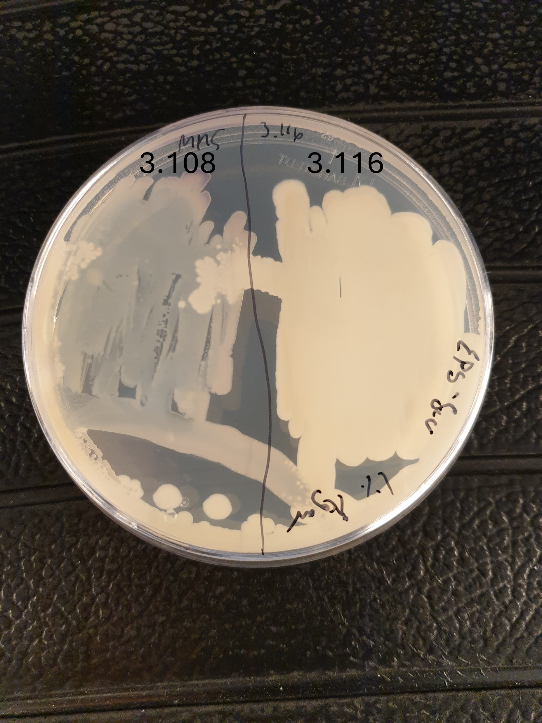

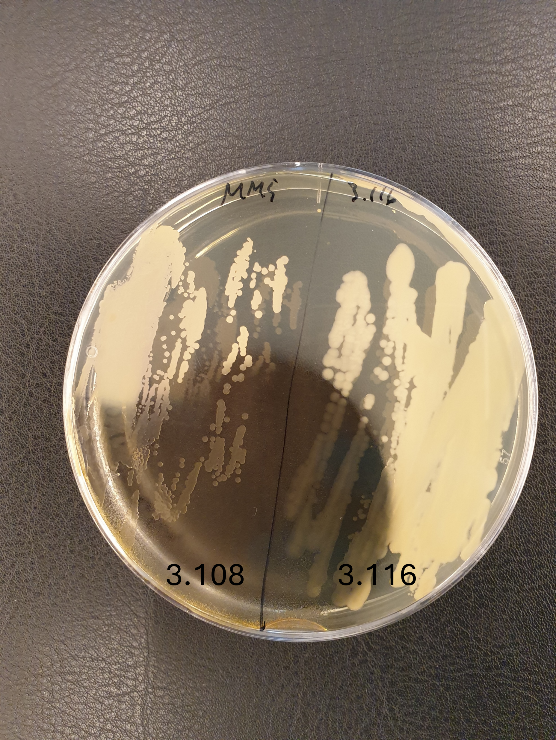


Figure S2: Agar plates with Janthinobacterium strains 3.108 and 3.116. A) EPS-sucrose with 1.5% agar incubated at 28°C. B) Strain 3.108 growing on LA-agar plates with 1% agar, incubated at 20°C. C) Both strains grown on LA-agar plates with 1% agar and 2% glycerol, incubated at 28°C D) Both strains grown on EPS-sucrose agar plates with 1% agar, incubated at 28°C. These results have previously been reported in a master thesis (Lorentsen 2020).


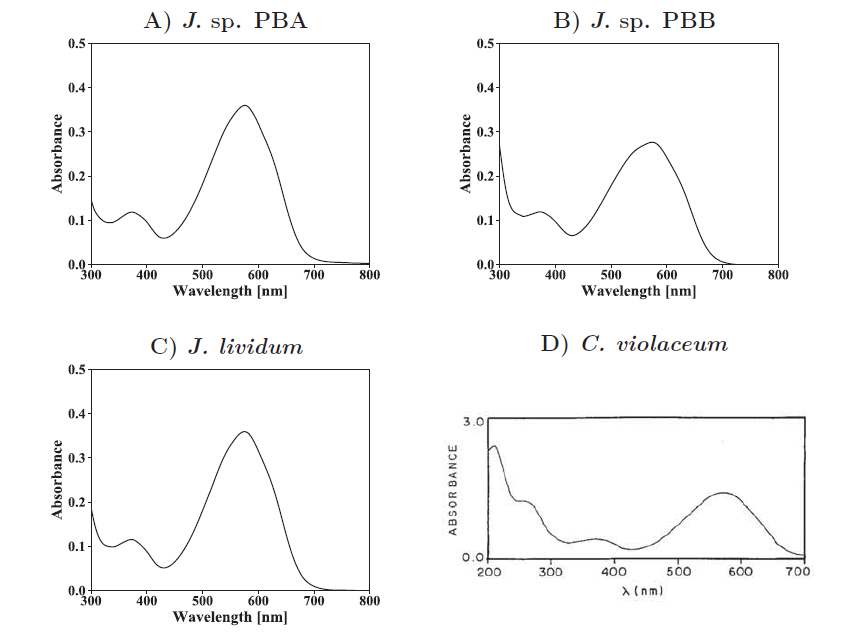


Figure S3: Absorption spectrums of violacein extracts from the strains A) pbA, B) pbB, and C) *Janthinobacterium lividum* type strain after cultivation for one week in liquid medium. The purple interface between air and medium was collected and dissolved in ethanol before determining the maximum absorbance. These results have previously been reported in a master thesis (Mølmen 2021). D) Absorption spectrum from *C. Violaceum* as measured by Rettori and Duran is included as a positive control (Rettori and Durán 1998).


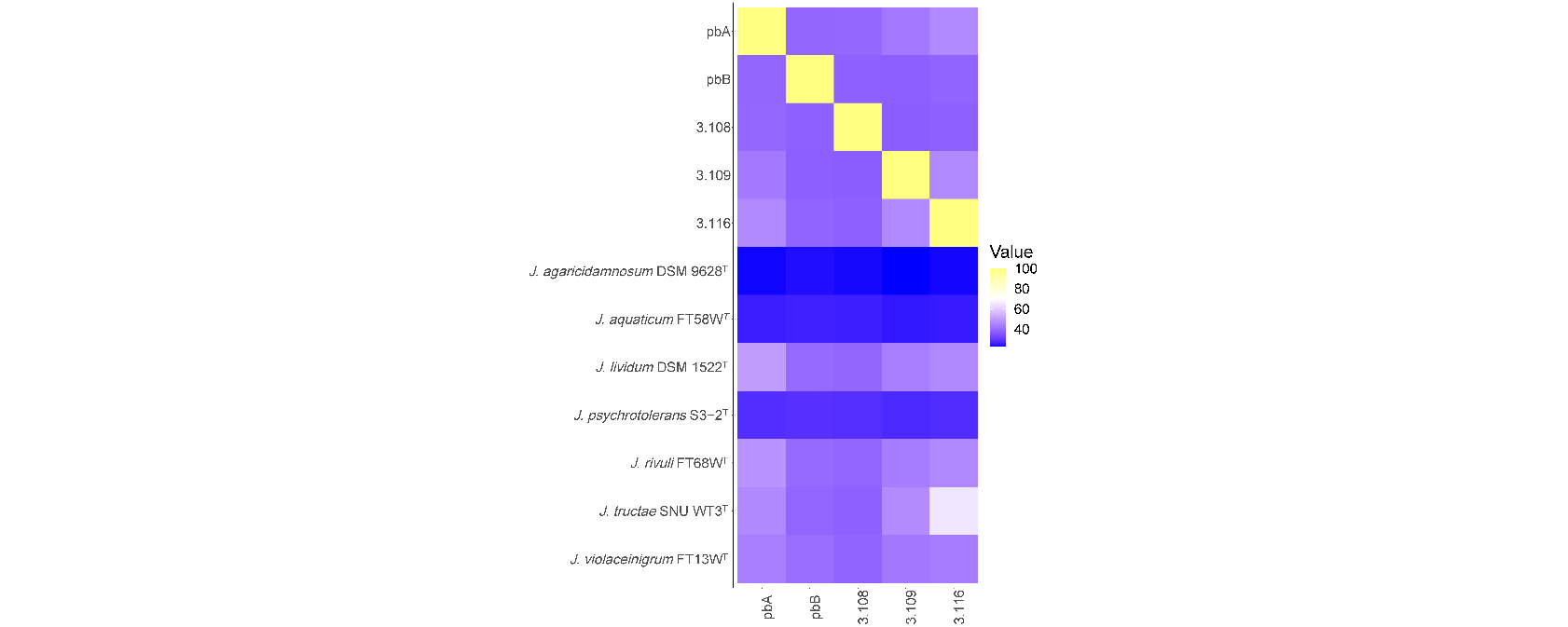


Figure S4: Heatmap showing the similarities of genome sequences based on isDDH analyses of the five *Janthinobacterium* strains reported in this study, and the seven *Janthinobacterium* type strains; *J. tructae* SNU WT3^T^, *J. lividum* DSM 1522^T^, *J. rivuli* FT68W^T^, *J. violaceinigrum* FT13W^T^, *J. psychrotolerans* S3-2^T^, *J. aquaticum* FT58W^T^, and *J. agaricidamnosum* DSM 9628^T^. The transition from blue to white (at 0.95) marks the threshold for species demarcation. Shades of yellow represent genomes assumed to be the same species.


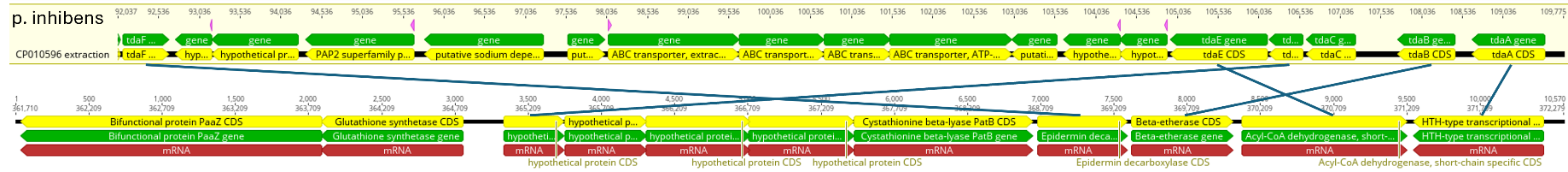


Figure S5: A schematic presentation of the alignment of the TDA operons of *Phaeobacter inhibens* strain P10 (Accession number: GCA_002888685.1) and the TDA operon of strain *Janthinobacterium sp.* 3.108. Geneious Prime 2023.0.1 ([https://www.geneious.com](http://www.geneious.com/)) was used to align the operons.


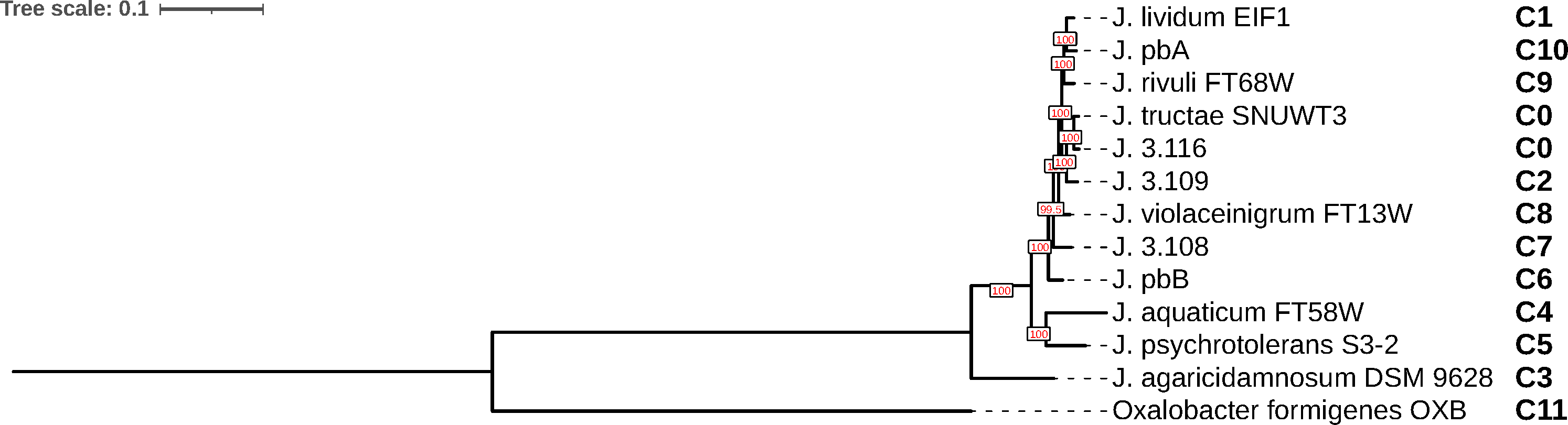

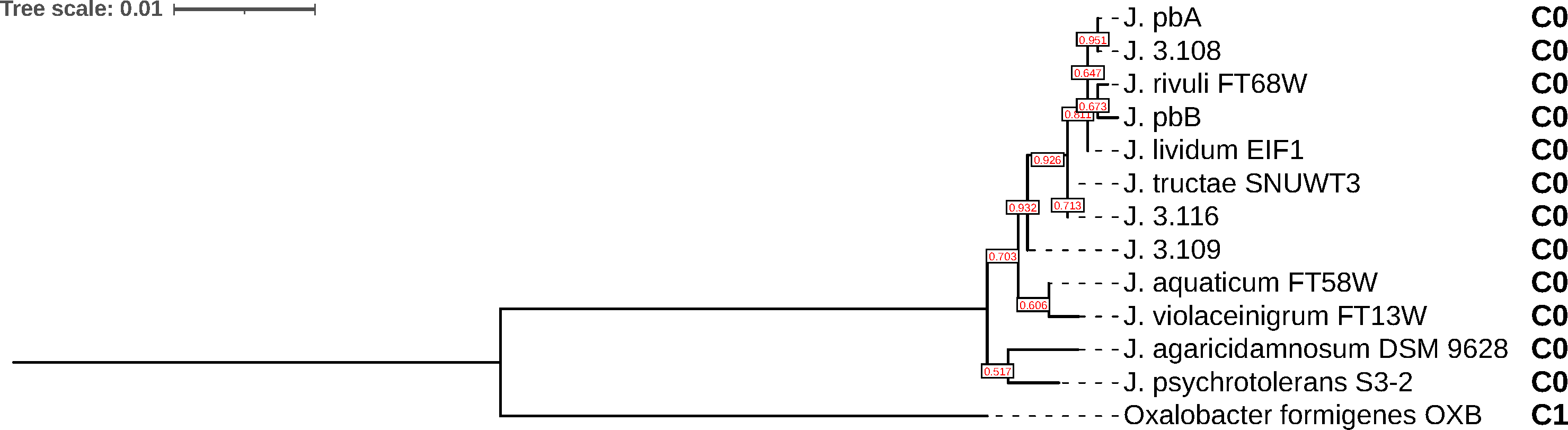


Figure S6: Phylogenetic Maximum likelihood trees constructed from A) 1060 orthologous core genes using the LG+F+I+G4 model, B) 16s rRNA gene sequences using Temura-Nei model for the five Janthinobacterium strains and seven Janthinobacterium-type strains. Oxalobacter formigens OXB was used as an outgroup. The numbers following the “C” represent species demarcation based on A) FastANI (Jain et al. 2018) and B) 97% identity for the 16s rRNA gene sequences.
