# Supplementary material for: Large genomic, functional, and phenotypical diversity of Janthinobacterium associated with Atlantic salmon fry
Source: FEMS Microbes. 2025 Oct 29;6:xtaf015. doi: 10.1093/femsmc/xtaf015 (PMC12618000; doi:10.1093/femsmc/xtaf015)
Supplement: xtaf015_Supplemental_Files [file xtaf015_supplemental_files.zip › FEMSMC-2025-006.R2 supplementary data - reviews and author response.docx]

**Reviews of manuscript FEMSMC-2025-006**

**Reviewer 1: Morten Dencker Schostag, Technical University of Denmark**

**Recommendation: Major Revision**

General comment:

This manuscript presents five Janthinobacterium strains isolated from Atlantic salmon fry and their rearing environments. The authors perform comparative genomic analysis, phenotypic assays, pangenome clustering, violacein production testing, and in vivo colonization experiments in gnotobiotic Atlantic salmon fry. The study is relevant to microbial ecology, fish microbiome research, and biotechnology. This manuscript presents solid and important work with appropriate methodology and scope for FEMS Microbes. With some major improvements, it will make a strong contribution.

Major concerns

The authors have done a nice job analyzing the five genomes and have done well with pangenomic analysis. However, the authors have mined the genomes for potential BGCs using antiSMASH but have not addressed the issue with the high numbers of contigs, there is almost a reverse correlation with the number of detected BGCs and the number of contigs (fig. 7). I highly recommend that the authors get all five strains nanopore sequenced to reduce the high number of contigs. To make comparative genomics and detection of BGC this is necessary to have ideally a closed genome or only a few contigs.

For the antimicrobial resistance assay, the authors have rightly used several antibiotics, however, there is no shown negative control to determine the right effect of the individual antibiotics. There should have been a susceptible strain in the antibiotic resistance assay.

For bioinformatics, the authors have not stated which parameters were used for each of the programs used, this makes the study less reproducible. The authors should provide a GitHub repository of the used bioinformatic pipeline or share it on an alternative repository platform.

Since mucin degradation potential on a genomic level was not detected in the five strains it would be necessary to determine degradation using chemical analysis and not only bacterial growth. Potential routes could be carbon hydrate analysis via LC/MS or NMR based monitoring of degradation.

Minor concerns

In figure 6 please have the same order of COG categories in the bar plot and the legend. I would also recommend changing the color hue so it is easier to distinguish them from each other.

In the colonization assay, how many fish survived in the different setups? Did the fish benefit from being colonized with the five Janthinobacterium strains? This could also be discussed more in detail to address the ecological role of the five Janthinobacterium strains.

Line 84: Which genes is the author referring to? Please specify which genes you mean.

Line 123: What does the author mean by satisfying? Please be more specific. Did you run a gel to check the DNA size distribution?

Line 142: Supplier information is missing for the M9 medium

Line 143: There is an extra parenthesis in this sentence

Line 195: It is unclear whether it is WT1 or WT3 for Janthinobacterium tructae SNU WT3 (Accession number: GCA_006517255.1). In Figure 4, it is labeled as WT1, while in the text it is referred to as WT3. Please ensure consistency.

Line 662-663: The numbers presented in the text do not match those in Figure 8. One of them appears to be incorrect.

Line 681: What do you mean by 'high diversity'? Given that all genomes in this study consist of multiple contigs, there appears to be insufficient information to support this claim. The authors should rephrase the sentence and clarify what is meant by 'high diversity'.

Line 686-687: The authors have compared the 16S rRNA gene from only five strains, which is insufficient to conclude that there is a low evolutionary rate of the 16S rRNA gene within the Janthinobacterium genus. To support such a statement, the analysis should include all Janthinobacterium strains used in Figure 4. This is highly recommended.

Line 777: Why JANTHINOBACTERIUM in capital letters? Please change it.

Line 779: “Had” is missing the sentence. It should be: “Wu et al. found that eight of 50 strains both had the AHL and JQS quorum sensing systems”

Line 808: I would have to disagree with that statement. Isolation of strain pbB from the wearing water could still originate from the skin of the fry, and vice versa for strain 3.108+3.109. How would the author distinguish the origin of the bacterial strains, whether it comes from the water or the skin?

Line 820: Again, what do the author mean with high diversity? Be more specific.

**Reviewer 2**

**Recommendation: Minor Revision**

In this manuscript, Lorentsen *et al*. investigate the significant genomic, functional, and phenotypical diversity of *Janthinobacterium* strains associated with Atlantic salmon fry. They hypothesized that these strains, despite similar 16S rRNA gene sequences, would exhibit considerable diversity and demonstrate varying abilities to colonize the salmon host. Their results reveal that the five isolated *Janthinobacterium* strains indeed represent distinct species with diverse phenotypic characteristics and a high potential for producing secondary metabolites. Furthermore, all strains successfully colonized Atlantic salmon yolk sac fry in gnotobiotic experiments, confirming their host-associated lifestyle. The conclusions drawn from this research are sound and justified, as they are well-supported by comprehensive genomic analyses, phenotypic characterization, and direct experimental evidence of host colonization.

This manuscript is clearly written, making the complex research accessible and understandable. The data is well-presented through various figures and tables, which effectively visualize the genomic, phylogenetic, and experimental findings, allowing for easy interpretation of the results. Overall, the study is robust, with only a few minor issues that would need addressing.

It would be beneficial for the authors to review the text for consistent use of capital letters, as they occasionally appear seemingly randomly or are used where not required (e.g., for chemicals, metabolites, or antibiotics). Similarly, consistent application of italic font for Latin words throughout the manuscript would further enhance its professional presentation.

In order of appearance:

Line 29: who or what ‘typically have antifungal properties'? Violacein or *Janthinobacterium* species?

Line 95: Perhaps the reason to use salmon fry as a source to isolate the bacteria, a sentence why salmon would be beneficial. Considering the authors are based in Norway, there might be a commercial reason to test salmon?

Line 123: ‘the quality was satisfying”. First of all, these is not a method but a result and secondly, this is rather qualitative and a quantitative argument would be better.

Lines 154-168: It is clear that, with hindsight perhaps, the experiments would have been done differently but the sentence at lines 158-159 does not look complete. Please rethink this paragraph and perhaps start with saying that originally a masters project looked at violacein production for strains 3.108 and 3.116, and the write the rest?

Line 177: Not sure if volume is useful in this context and amount in micro gram would be more informative.

Lines 291-294: This is results, not methods, and better explained in the results section perhaps.

Line 393: The reader is referred to Table 1 regarding the more raised colony feature of pbB but in that table all strains apparently have a raised phenotype?

Figure 2: Please use a more contrasting font colour as the black against the dark background is very difficult to read.

Line 486: It is mentioned that “the core genomes encompassed of 34,872 genes” which I find somewhat confusing. Perhaps I am misunderstanding but as the individual genomes have between 5500 and about 6000 genes, how can the core genome be more than any individual genome? The core genome is comprised of the genes all species in the collection share and therefor always less than the number of genes in the organism with the lowest gene count?

Line 777: Please change the shouty JANTHINOBACTERIUM to correct format.

Line 832: The authors might want to link the presence of the AHL QS system to violacein? Their findings reinforce the hypothesis that this system is actually linked to the dye?

References: Please provide more information for the master theses, assuming they are in the university or national register with more identifying information.

**Associate Editor Recommendation: Major Revision**

Thank you, first of all, for the pleasure of reading your manuscript and for your understanding with the exceptionally long amount of time the manuscript was under review. It was a challenging season to find reviewers and I sincerely thank you for your patience.

Based on the feedback of the two peer reviewers, I would suggest a major revision including the changes listed by the reviewers. Changes of note include making the code and parameters used for bioinformatics analyses available, showing negative control data if possible for the antibiotic assays, and addressing the issue in the text of a high number of contigs resulting in an almost reverse correlation with the number of detected BGCs and the number of contigs (Fig. 7). One reviewer suggested that the strains should be Nanopore sequenced – if this is possible within your project and capabilities then it would of course greatly strengthen the manuscript. However, I recognize this is significant additional effort and expense. The issue could alternately be addressed in the Discussion of the limitations of the fragmented genomes with many contigs to the and revisiting fragmented BGCs for closer analysis. With the revisions suggested by the reviewers in place, the manuscript, already strong, will be greatly strengthened.

**EIC Decision: Major Revision**

**Author's Response to Decision Letter for (FEMSMC-2025-006)**

Rev.1

Major concerns

The authors have done a nice job analyzing the five genomes and have done well with pangenomic analysis. However, the authors have mined the genomes for potential BGCs using antiSMASH but have not addressed the issue with the high numbers of contigs, there is almost a reverse correlation with the number of detected BGCs and the number of contigs (fig. 7). I highly recommend that the authors get all five strains nanopore sequenced to reduce the high number of contigs. To make comparative genomics and detection of BGC this is necessary to have ideally a closed genome or only a few contigs.

Thank you for your suggestion. We agree that it would be beneficial to do nanopore sequencing of the strains to reduce the number of contigs, especially to improve the confidence in the BGC analyses. However, due to limited resources in this project (time, manpower, financial), resequencing and reanalysis of the genomes would be challenging to us. We still hope that the manuscript can be published in FEMS Microbes. We have revised the text to clarify the limitations of our genome sequencing data in the manuscript (see details below). We think that the comparative genomic analyses (FastANI, isDDH from TYGS, and phylogenetic tree) remain valid, even though we only have contig-level genomes. The genomes are (according to CheckM) more than 99% complete, and according to the authors behind FastANI (https://doi.org/10.1038/s41467-018-07641-9), PubMLST (10.1099/mic.0.055459-0 ) and isDDH (https://doi.org/10.1186/1471-2105-14-60) these methods should be quite robust to incomplete genomes. When it comes to the phylogenetic tree, only core genes that are present in all the genomes was used for the analyses. However, we agree that the pangenome, antiSMASH and prophage analyses may be affected by the relative large number of contigs. We have adressed this issue in the discussion, see line 634-651. For the pangenome analyses, incomplete genomes might result in wrong estimation of the number of genes due to missing genes and fragmented genes, this could alter the proportions of core genes, singletons, etc. We have now pointed this out in line 241-244, 641-651, and Figure text of Fig. 5 (line 1123-1125). When it comes to the analyses of secondary metabolite gene clusters, the incomplete genomes and presence of several contigs, could result in overestimation or underestimation of the number of BGCs. Underestimation could harbour from lack of genes due to incomplete sequences, while an overestimation would likely originate from gene-fragments on the edges of the contigs being counted multiple times. We checked this potential overestimation in our data, and the only strain where this occurred for was strain pbB, where one group of BGCs (NRPS) was found on the edge of two different contigs. We have pointed this out in lines 502-506, 692-696, and Figure text of Fig. 7 (line 1144-1145). Furthermore, repeating parts of the sequences, such as mobile genetic elements, are usually underrepresented. This could lead to an underrepresentation of the number of phage sequences. We have pointed this out in lines 646-649.

For the antimicrobial resistance assay, the authors have rightly used several antibiotics, however, there is no shown negative control to determine the right effect of the individual antibiotics. There should have been a susceptible strain in the antibiotic resistance assay.

We agree, and have now repeated these experiments, using E.coli K12 MG1655 as a susceptible strain. See line 183, and Table S10

For bioinformatics, the authors have not stated which parameters were used for each of the programs used, this makes the study less reproducible. The authors should provide a GitHub repository of the used bioinformatic pipeline or share it on an alternative repository platform.

Thank you for reminding us of this. We have now uploaded the script for the pangenome and phylogenetic analyses to GitHub. We included a reference to this in the Methods section, line 210-211 and 221-222. Most of the remaining analyses were done through the respective web-platforms using default parameters (Line 188-189, 241, 252-254, and 261).

Since mucin degradation potential on a genomic level was not detected in the five strains it would be necessary to determine degradation using chemical analysis and not only bacterial growth. Potential routes could be carbon hydrate analysis via LC/MS or NMR based monitoring of degradation.

We agree that it would be very interesting to study the mucin degradation further and to include this kind of analysis, but unfortunately it is not currently feasible for us, and we feel that it is a bit beyond the scope of this study. We hope to be able to follow up this in later studies.

Minor concerns

Figures:

Figure 6: In figure 6 please have the same order of COG categories in the bar plot and the legend. I would also recommend changing the color hue so it is easier to distinguish them from each other.

We agree and have changed the figure such that the order in the bar plot and legend is the same. We also made a more contrasting colour palette.

In the colonization assay, how many fish survived in the different setups? Did the fish benefit from being colonized with the five Janthinobacterium strains? This could also be discussed more in detail to address the ecological role of the five Janthinobacterium strains.

We have now addressed mortality in the results, line 558-559. During these two experiments, only 1 fish died. Generally, yolk sac fry of Atlantic salmon is rather robust, and to evaluate performance, we would probably need to do analyses at the gene expression level. Again, this was beyond the scope of this study.

Introduction

Line 84: Which genes is the author referring to? Please specify which genes you mean.

We have now elaborated this in the text, see line 83-85.

Materials and Method

Line 123: What does the author mean by satisfying? Please be more specific. Did you run a gel to check the DNA size distribution?

This has now been clarified in the text (line 125-127).

Line 142: Supplier information is missing for the M9 medium

Thank you, it is now corrected (line 146).

Line 143: There is an extra parenthesis in this sentence

Thanks for pointing out, it is now removed

Line 195: It is unclear whether it is WT1 or WT3 for Janthinobacterium tructae SNU WT3 (Accession number: GCA_006517255.1). In Figure 4, it is labeled as WT1, while in the text it is referred to as WT3. Please ensure consistency.

Thank you for pointing this out. We have now ensured consistency by correcting to WT3 in Figure 4

Results

Line 662-663: The numbers presented in the text do not match those in Figure 8. One of them appears to be incorrect.

This is due to the numbers in the text being averages, while the boxes representing median ± interquartile range. See lines 576-577 and Figure text of fig. 8 (line 1154-1155).

Discusion

Line 681: What do you mean by 'high diversity'? Given that all genomes in this study consist of multiple contigs, there appears to be insufficient information to support this claim. The authors should rephrase the sentence and clarify what is meant by 'high diversity'.

By high diversity, we mean high phenotypic, genomic and functional diversity. We have clarified this in the manuscript (line 584-585). We still believe that our analyses of colony phenotypes (Table 1, Fig.1), genomic differences (FastANI, isDDH, phylogenomics, pangenome), and potential for secondary metabolite production (from Antismash) demonstrate that the strains are diverse in these respects. Furthermore, as pointed out above, both the FastANI and the isDDH analyses used in this study should be quite robust to errors due to incomplete genomes, according to the authors.

Line 686-687: The authors have compared the 16S rRNA gene from only five strains, which is insufficient to conclude that there is a low evolutionary rate of the 16S rRNA gene within the Janthinobacterium genus. To support such a statement, the analysis should include all Janthinobacterium strains used in Figure 4. This is highly recommended

Thanks for your suggestion. To provide further evidence we of this statement, we have now compared the phylogenetic trees based on core genes and 16s rRNA genes, average nucleotide identity for the genomes and percent identity of our five strains and the seven type strains (See line 590-600, Table S15, and Fig S6).

Line 777: Why JANTHINOBACTERIUM in capital letters? Please change it.

Corrected, see line 718-719

Line 779: “Had” is missing the sentence. It should be: “Wu et al. found that eight of 50 strains both had the AHL and JQS quorum sensing systems”

Corrected, see line 720.

Line 808: I would have to disagree with that statement. Isolation of strain pbB from the wearing water could still originate from the skin of the fry, and vice versa for strain 3.108+3.109. How would the author distinguish the origin of the bacterial strains, whether it comes from the water or the skin?

We agree, and have revised the text accordingly (line 751-755)

Conclusion

Line 820: Again, what do the author mean with high diversity? Be more specific.

We have revised the text, and hope that it is clearer now (line 766-767)

Rev. 2

It would be beneficial for the authors to review the text for consistent use of capital letters, as they occasionally appear seemingly randomly or are used where not required (e.g., for chemicals, metabolites, or antibiotics). Similarly, consistent application of italic font for Latin words throughout the manuscript would further enhance its professional presentation.

Thank you for pointing this out, we have gone through the manuscript and corrected these mistakes.

Abstract

Line 29: who or what ‘typically have antifungal properties'? Violacein or Janthinobacterium species?

It should be Janthinobacterium. We have clarified this now (line 29)

Line 95: Perhaps the reason to use salmon fry as a source to isolate the bacteria, a sentence why salmon would be beneficial. Considering the authors are based in Norway, there might be a commercial reason to test salmon?

This study was based on previous research activities and a collection of bacterial isolates from salmon fry maintained in our research group. We now clarified this in the text (line 96-97).

Material & Methods

Line 123: ‘the quality was satisfying”. First of all, these is not a method but a result and secondly, this is rather qualitative and a quantitative argument would be better.

We have now clarified how the quality of the DNA was assessed (line 125-127). We agree that this could be considered as results, however, we feel that this is very basic information that matters mainly for the further genome sequencing. We therefore hope that we can keep this information to the Methods section.

Lines 154-168: It is clear that, with hindsight perhaps, the experiments would have been done differently but the sentence at lines 158-159 does not look complete. Please rethink this paragraph and perhaps start with saying that originally a masters project looked at violacein production for strains 3.108 and 3.116, and the write the rest?

Thank you for pointing this out. We have revised this paragraph and hope this is now more clearly presented (line 158-176).

Line 177: Not sure if volume is useful in this context and amount in micro gram would be more informative.

Thank you for pointing this out, we agree that the volume alone is not so useful, we have now included concentrations and volume see line 185-186.

Lines 291-294: This is results, not methods, and better explained in the results section perhaps.

Thank you for your suggestion, we agree that this also belongs in the result section, it is now described in the figure text of Fig. 8 line 1160-1165. However, we also think it’s natural to explain in the methods section (line 319-323)

Results

Line 393: The reader is referred to Table 1 regarding the more raised colony feature of pbB but in that table all strains apparently have a raised phenotype?

Thank you for pointing this out, it should only be referred to Figure 1A here. The text is corrected accordingly (line 381)

Figure 2: Please use a more contrasting font colour as the black against the dark background is very difficult to read.

Thank you for pointing this out, we have now changed the background colour to white.

Line 486: It is mentioned that “the core genomes encompassed of 34,872 genes” which I find somewhat confusing. Perhaps I am misunderstanding but as the individual genomes have between 5500 and about 6000 genes, how can the core genome be more than any individual genome? The core genome is comprised of the genes all species in the collection share and therefor always less than the number of genes in the organism with the lowest gene count?

This is the total number of core genes across all the strains. We agree that it could be confusing, thus we changed it to number of genes (line 444).

Discussion

Line 777: Please change the shouty JANTHINOBACTERIUM to correct format.

Thanks for pointing out, corrected (lines 718-719)

Conclusion

Line 832: The authors might want to link the presence of the AHL QS system to violacein? Their findings reinforce the hypothesis that this system is actually linked to the dye?

Thank you for your suggestion, added a sentence about this in the conclusion (lines 781-782). Furthermore, we added a sentence about this in the discussion (line 723-724)

References

Please provide more information for the master theses, assuming they are in the university or national register with more identifying information.

We have now added links to the archive where these can be accessed.

**Reviews of manuscript FEMSMC-2025-006.R1**

**Reviewer 1: Morten Dencker Schostag, Technical University of Denmark**

**Recommendation: Accept**

I would like to thank the authors for carefully considering my comments and suggestions. I believe they have thoroughly and satisfactorily addressed the concerns raised in the previous round of review. The revisions have improved the clarity and quality of the manuscript. I have no further major concerns, and I recommend the manuscript for publication in its current form.

**Reviewer 2**

**Recommendation: Minor Revision**

Only minor comments, am satisfied with the authors’ responses to my earlier comments.

Line 28-29: “They are known for producing violacein, and they typically have antifungal properties.” This could be more clear: “They are known for producing violacein which has antifungal properties.”

Line 65: B. dendrobatidis (not full genus name any longer).

Line 77: However, Haack et al. found that the antifungal properties of Janthinobacterium were not primarily linked to violacein but found that their activity was reduced by knocking out the Janthinobacterium quorum sensing system (Haack et al. 2016).

Their activity not completely clear? The activity of violacein? Or antifungal properties? Is it meant that Janthinobacterium antifungal properties are reduced when quorum sensing is affected? This can be rewritten to be less ambiguous.

Line 88: It is similar to the quorum sensing systems found in Vibrio cholerae and Legionella pneumophila, which are involved in regulating host-pathogen interactions (Tiaden and Hilbi 2012; Simon et al. 2015).

Line 100: I assume this is flask (not flakes) as flaks is probably wrong?

Line 206/7: Anvi’o

Line 233 and whole manuscript: 16S

Line 258: and the Comprehensive Antibiotic Resistance Database (CARD) (CARD 2023: Expanded Curation, Support for Machine Learning, and Resistome Prediction at the Comprehensive Antibiotic Resistance Database - PubMed) were used to screen the genomes for antibiotic resistance genes…

Please properly cite this:

Alcock BP, Huynh W, Chalil R, Smith KW, Raphenya AR, Wlodarski MA, Edalatmand A, Petkau A, Syed SA, Tsang KK, Baker SJC, Dave M, McCarthy MC, Mukiri KM, Nasir JA, Golbon B, Imtiaz H, Jiang X, Kaur K, Kwong M, Liang ZC, Niu KC, Shan P, Yang JYJ, Gray KL, Hoad GR, Jia B, Bhando T, Carfrae LA, Farha MA, French S, Gordzevich R, Rachwalski K, Tu MM, Bordeleau E, Dooley D, Griffiths E, Zubyk HL, Brown ED, Maguire F, Beiko RG, Hsiao WWL, Brinkman FSL, Van Domselaar G, McArthur AG. CARD 2023: expanded curation, support for machine learning, and resistome prediction at the Comprehensive Antibiotic Resistance Database. Nucleic Acids Res. 2023 Jan 6;51(D1):D690-D699. doi: 10.1093/nar/gkac920.

Line 267: Endawoke is first name? Please check all citations/references for simple things like this.

Line 361: What is MM5 in figure 2? Isolate 3.108?

Line 391: All strains showed resistance towards ampicillin (100 µg/ml) and streptomycin (25 µg/ml), even though we observed some growth for strain 3.116 when exposed to ampicillin (100 µg/ml) and for pbA and 3.116 when exposed to streptomycin (25 µg/ml).

This seems confusing? All strains are resistant but 3.166 and pbA show some growth? So not that resistant as the others? This needs clarifying.

**Associate Editor Recommendation: Accept**

The authors have responded to the reviewers comments and greatly improved the text. There are a few typos which were caught by one reviewer which appear to be fair and worth integrating into the text prior to publication but they are minor changes which does not require another round with the reviewers. As an additional small fix, line 75: N-acetyl-d-glucosamine typically should be written with an italicized 'N'.

**EIC Decision: Minor Revision**

**Author's Response to Decision Letter for (FEMSMC-2025-006.R1)**

Associate Editor

Comments to the Author:

The authors have responded to the reviewers comments and greatly improved the text. There are a few typos which were caught by one reviewer which appear to be fair and worth integrating into the text prior to publication but they are minor changes which does not require another round with the reviewers. As an additional small fix, line 75: N-acetyl-d-glucosamine typically should be written with an italicized 'N'.

Thank you for pointing this out, it is now fixed, see line 79

Reviewer: 2

Comments to the Author

Only minor comments, am satisfied with the authors’ responses to my earlier comments.

Line 28-29: “They are known for producing violacein, and they typically have antifungal properties.” This could be more clear: “They are known for producing violacein which has antifungal properties.”

Thank you for suggesting this revision, however, as Haack et al (2016) (https://doi.org/10.3389/fmicb.2016.01668) suggest that the antifungal properties of Janthinobacterium are not primarily linked with the synthesis of violacein, and we therefore disagree with this suggestion.

Line 65: B. dendrobatidis (not full genus name any longer).

Corrected, line 64-65

Line 77: However, Haack et al. found that the antifungal properties of Janthinobacterium were not primarily linked to violacein but found that their activity was reduced by knocking out the Janthinobacterium quorum sensing system (Haack et al. 2016).

Their activity not completely clear? The activity of violacein? Or antifungal properties? Is it meant that Janthinobacterium antifungal properties are reduced when quorum sensing is affected? This can be rewritten to be less ambiguous.

Thank you for pointing this out. We agree that this was written quite ambiguous. We have addressed this in line 77-79

Line 88: It is similar to the quorum sensing systems found in Vibrio cholerae and Legionella pneumophila, which are involved in regulating host-pathogen interactions (Tiaden and Hilbi 2012; Simon et al. 2015).

Thank you, corrected in line 88-89

Line 100: I assume this is flask (not flakes) as flaks is probably wrong?

Corrected, line 100

Line 206/7: Anvi’o

Corrected, line 205

Line 233 and whole manuscript: 16S

Thanks for pointing this out, it is now fixed throughout the manuscript

Line 258: and the Comprehensive Antibiotic Resistance Database (CARD) (CARD 2023: Expanded Curation, Support for Machine Learning, and Resistome Prediction at the Comprehensive Antibiotic Resistance Database - PubMed) were used to screen the genomes for antibiotic resistance genes…

Please properly cite this:

Alcock BP, Huynh W, Chalil R, Smith KW, Raphenya AR, Wlodarski MA, Edalatmand A, Petkau A, Syed SA, Tsang KK, Baker SJC, Dave M, McCarthy MC, Mukiri KM, Nasir JA, Golbon B, Imtiaz H, Jiang X, Kaur K, Kwong M, Liang ZC, Niu KC, Shan P, Yang JYJ, Gray KL, Hoad GR, Jia B, Bhando T, Carfrae LA, Farha MA, French S, Gordzevich R, Rachwalski K, Tu MM, Bordeleau E, Dooley D, Griffiths E, Zubyk HL, Brown ED, Maguire F, Beiko RG, Hsiao WWL, Brinkman FSL, Van Domselaar G, McArthur AG. CARD 2023: expanded curation, support for machine learning, and resistome prediction at the Comprehensive Antibiotic Resistance Database. Nucleic Acids Res. 2023 Jan 6;51(D1):D690-D699. doi: 10.1093/nar/gkac920.

Thanks for pointing this out, it is fixed in line 255 and 1209

Line 267: Endawoke is first name? Please check all citations/references for simple things like this.

Corrected, line 271

Line 361: What is MM5 in figure 2? Isolate 3.108?

Thanks for pointing this out, should be 3.108, it is now fixed

Line 391: All strains showed resistance towards ampicillin (100 µg/ml) and streptomycin (25 µg/ml), even though we observed some growth for strain 3.116 when exposed to ampicillin (100 µg/ml) and for pbA and 3.116 when exposed to streptomycin (25 µg/ml).

This seems confusing? All strains are resistant but 3.166 and pbA show some growth? So not that resistant as the others? This needs clarifying.

Thank you for pointing this out, we agree that it’s confusing and have tried to clarify – see line 386-387

**Reviews of manuscript FEMSMC-2025-006.R2**

**Associate Editor Recommendation: Accept**

Thank you for making these further changes to the text suggested by reviewer 2. The comments have been adequately addressed.

**EIC Decision: Accept**
